# Assessment of the influence of Nutri-Score on Polish consumer Choices - Insights from the nationwide, Cross-sectional study

**DOI:** 10.1038/s41598-025-14033-9

**Published:** 2025-08-11

**Authors:** Hubert Dobrowolski, Dariusz Włodarek, Mariusz Panczyk

**Affiliations:** 1 School of Medical & Health Sciences, University of Economics and Human Sciences in Warsaw, Warsaw, 01-043 Poland; 2 Department of Dietetics, Institute of Human Nutrition Sciences, Warsaw University of Life Sciences (SGGW), Warsaw, 02-776 Poland; 3 Department of Education and Research in Health Sciences, Faculty of Health Science, Medical University of Warsaw, Warsaw, 00-581 Poland

**Keywords:** Disease prevention, Nutrition, Patient education, Public health

## Abstract

The public is increasingly looking for healthier alternatives in food products. Front-of-pack labelling aims to help consumers make healthier food choices. The aim of our study was to investigate whether Nutri-Score can improve nutritional choices of Polish consumers. The survey was conducted using the CAWI method. The proprietary questionnaire included metric questions, knowledge about the Nutri-Score system and questions on nutritional knowledge from the KomPAN questionnaire. To assess the effectiveness of the Nutri-Score system, participants compared healthiness of 8 product pairs with visible and invisible Nutri-Score labelling. The study involved 1035 participants, a representative sample of the Polish population. Participants had average nutritional knowledge (5.6 ± 2.14 out of 10 points) and average knowledge of Nutri-Score (2.9 ± 1.66 out of 7 points). The results showed that visibility of the system did not significantly (*p* > 0.05) influence consumers’ dietary choices. An unfavourable dietary change was shown (*p* < 0.001) for two pairs of products: fish (OR of 11.56 (95% CI [8.40–15.91]) and ham OR of 2.84 (95% CI [2.19–3.69]). The distribution of the data obtained indicated a negative effect of the system on consumer choices. In conclusion, our study showed that the Nutri-Score system has a limited effect on improving health-promoting dietary choices.

**Supplementary Information:**

The online version contains supplementary material available at 10.1038/s41598-025-14033-9.

## Introduction

Diet and lifestyle undoubtedly have an effect on the health of the population. With the increasing percentage of people with excess body mass, including obesity, and the consequent increase in the risk of major noncommunicable diseases, increasing amounts of time and financial resources are being devoted to the prevention of obesity and diseases resulting from excess body mass^1–3^. Front-of-pack labelling (FOPL) is one tool created to improve the diet of the population.

FOPL is a simplified and easy-to-understand presentation of a food’s nutritional information^4,5^, designed to help consumers make the right purchasing decision^6^. The World Health Organization has indicated that these schemes are important tools for promoting healthy diets and raising consumer awareness^7^. Although this position is supported by the results of previous studies^8^, some studies point to ambiguity regarding the effectiveness of FOPL systems^9^. It is also pointed out that while the FOPL may translate into a better understanding of the health of a product, it does not necessarily mean that it results in healthier behaviours or dietary choices^10^.

One system of particular interest in recent years is the Nutri-Score system developed in France, which belongs to the group of interpretative systems. Its algorithm considers the selected components of a product’s nutritional value, giving it a final score on a colour scale from dark green to dark orange and on a letter scale from A to E^11^. The components of the algorithm and the calculation methods have changed several times over the years^12,13^. As a result of the critical work demonstrating the shortcomings of the system, developers have made several corrections to improve it. In its current form, in the latest version, the system scores beverages, fats, and other foods separately (with cheese scored slightly differently), considering components such as the energy value of the product; the sugar, salt, saturated fatty acid, dietary fibre, and protein content (of which red meat may receive a limited number of points compared to other products); the percentage of fruits, vegetables, and legumes; and the presence of selected sweeteners^14^.

Several studies have assessed the potential impact of the Nutri-Score system on consumer food choices. Studies have shown the superior effectiveness of the Nutri-Score system in informing consumers about the nutritional quality of products compared with that of other labels, which may result in improved dietary choices and reduced risk of chronic diseases^15–19^. Akker et al. (2021) showed that participants made healthier dietary choices using the Nutri-Score system^20^. On the other hand, Andreeva et al. (2021) showed an improvement in consumers’ ability to classify foods according to nutritional quality, nutritional quality of actual and planned food purchases, and portion size choices^21^. However, numerous studies demonstrating the effectiveness of the system were conducted before the latest update. In addition, the vast majority of these studies were conducted by developers of the system, which could be considered a conflict of interest in the studies performed^22,23^. Other papers, in turn, demonstrating that the Nutri-Score system does not function correctly, were questioned in the work of Besancon et al. (2023), which shows that studies with declared conflicts of interest are substantially more likely to report negative outcomes regarding Nutri-Score (adjusted OR = 21)^24^.

Given the significance of FOPL systems for public health and their potential to enhance diet quality, coupled with the lack of independent studies assessing the effectiveness of the latest version of the Nutri-Score algorithm and the absence of research involving a representative sample of Polish consumers, the aim of our study was to determine whether the Nutri-Score system improves the consumer’s ability to recognise the nutritionally preferable product.

## Results

### Characteristics of study participants

The survey included 1,035 participants with equal sex distribution and diverse educational backgrounds. Most participants were actively employed, with the largest age group being 35–44 years. The size of the participants’ places of residence were widely distributed, demonstrating a broad geographic spread across various provinces in Poland. Financially, over half reported having enough income for daily expenses but not for larger purchases. Shopping habits varied, with most participants shopping alone or with their family members. Nearly one-third of the households included children under 13 years of age, and approximately 10% of the respondents required a special diet for chronic illnesses. Detailed socioeconomic and demographic characteristics of the participants are presented in Table 1.

**Table 1:** Socio-Demographic and economic profile of study Participants.

Check	*N*	%
**Sex** Female Male	528 507	51.01 48.99
**Education** primary or lower secondary school basic vocational/professional secondary, post-secondary higher	126 245 377 287	12.17 23.67 36.43 27.73
**Age group** 18–24 years old 25–34 years old 35–44 years old 45–54 years old 55–64 years old 65 years old and more	87 168 232 199 173 176	8.41 16.23 22.42 19.23 16.71 17.00
**Size of place of permanent residence** rural areas small town (less than 20 000 inhabitants) city of 20 001 to 100 000 inhabitants city of 100 001 to 200 000 inhabitants city of more than 200 001 inhabitants	414 140 193 93 195	40.00 13.53 18.65 8.99 18.84
**Employment situation** I am not working and studying/learning I am a full-time housekeeper I work I am studying/learning I prefer not to answer this question	157 93 682 72 31	15.17 8.99 65.89 6.96 3.00
**Financial situation** I prefer not to answer this question Not enough money even for immediate needs We have to refuse ourselves many things in order to have enough money for basic needs - food, hygiene products There is enough money on a daily basis, but we cannot afford to spend more There is enough money for all expenses and we can save some of it We are wealthy, we do not need to save even for major expenses	23 39 116 543 283 31	2.22 3.77 11.21 52.46 27.34 3.00
**Special diet in connection with a chronic disease** No Yes No chronic disease	161 104 770	15.56 10.05 74.40

### Assessment of nutritional knowledge among study participants

The average score achieved in the nutritional knowledge test was 5.6 ± 2.14 out of a possible 10 points, with a median score of 6.0. Notably, participants struggled with three questions, with over 75% being unable to provide correct answers, whereas for six questions, more than 70% of the respondents answered correctly. Further details on participants’ responses are presented in Table 2.

**Table 2:** Distribution of correct responses in the Nutritional Knowledge Test.

No	Statement	Number of correct answers	
		*N*	%
1	It is sufficient to consume cereal products once a day.	255	24.64
2	Fruits and/or vegetables should be consumed with every meal.	724	69.95
3	High salt intake protects against hypertension.	773	74.69
4	Reducing fatty foods in the diet helps prevent cardiovascular diseases.	845	81.64
5	Bio-yoghurts contain beneficial intestinal bacteria.	788	76.14
6	Oil and olive oil contain a lot of cholesterol.	502	48.50
7	Wholemeal bread contains more fibre than white bread.	798	77.10
8	Fruits and vegetables are a source of “empty calories”.	748	72.27
9	Yellow cheese is a better source of calcium than cottage cheese.	228	22.03
10	Protein should be the main source of energy in a proper diet.	145	14.01

### Assessment of knowledge of Nutri-Score system among study participants

The average score obtained by respondents in the Nutri-Score labelling knowledge test was 2.9 ± 1.66 (median 3.0) out of a possible 7 points. Table 3 presents the structure of responses from the study group. The highest number of correct answers was obtained by respondents with visual aspects of labelling, i.e. colour gradation (80.03%) and alphabetical gradation (66.57%).

**Table 3:** Knowledge of the Nutri-Score labelling system.

No	Question	% of responses given	
		Correct	Incorrect /”don’t know”
1	Does Nutri-Score use a grading/colour scale (red to green) to assess the nutritional value of foods?	80.03	19.97
2	Does Nutri-Score use a graded numerical scale (1 to 5) to assess the nutritional value of food?	33.72	66.28
3	Does Nutri-Score use a graded alphabetical scale (E to A) to assess the nutritional value of foods?	66.57	33.43
4	Can Nutri-Score be used to compare the nutritional value between two products belonging to the same category (e.g. cheese with another cheese or juice with another juice)?	57.74	42.26
5	Can Nutri-Score be used to compare the nutritional value between two products belonging to different categories (e.g. cheese with juice or bread with cold cuts)?	20.55	79.45
6	Does Nutri-Score have another, separate designation for dangerous products?	16.50	83.50
7	Does the Nutri-Score system rate food products differently, i.e. cheeses, beverages, baked goods, and cured meats?	18.23	81.77

### Nutri-Score label visibility and consumer food choices

The analysis highlights that Nutri-Score label visibility does not significantly affect consumer opinion on the healthiness of the productacross most food categories. However, two categories, fish and hams, exhibited notable shifts towards less favourable choices when the Nutri-Score labels were visible. These changes were statistically significant, with fish showing OR of 11.56 (95% CI [8.40–15.91]) and hams an OR of 2.84 (95% CI [2.19–3.69]), both indicating a significant influence of label visibility on consumer opinion. An OR greater than 1 indicates an increased likelihood of less favourable choices due to label visibility, demonstrating the significant influence of the Nutri-Score system on consumer decisions in these categories (Table 4).

**Table 4:** Association between Nutri-Score label visibility and consumer choice by food category.

No	Product category	Percent change in choice towards a less favourable product	Percent change in choice towards a more favourable product	χ^2^	*P*-value*	OR [95% CI]
1	Yoghurts	9.57	7.15	3.329	0.068	1.33 [0.99–1.79]
2	Breads	12.17	11.11	0.415	0.519	1.09 [0.85–1.40]
3	Pastas	11.21	8.60	3.298	0.069	1.33 [1.00–1.74]
4	Drinks	8.02	8.60	0.145	0.703	0.97 [0.72–1.30]
5	Cereals	7.54	9.18	1.480	0.224	0.85 [0.64–1.15]
6	Herrings	8.12	8.60	0.093	0.761	0.92 [0.68–1.26]
7	Fish	18.74	6.09	65.759	< 0.001	11.56 [8.40–15.91]
8	Hams	25.22	12.95	40.192	< 0.001	2.84 [2.19–3.69]

This study demonstrates that the visibility of Nutri-Score labels does not significantly influence consumer opinion on the healthiness of the productin most food categories (*p* > 0.05). These results suggest general neutrality or a lack of responsiveness to labels across these product categories. However, the visibility of Nutri-Score labels significantly influenced consumer opinion in two specific food categories: fish and ham (*p* < 0.001).

**Fig. 1:**
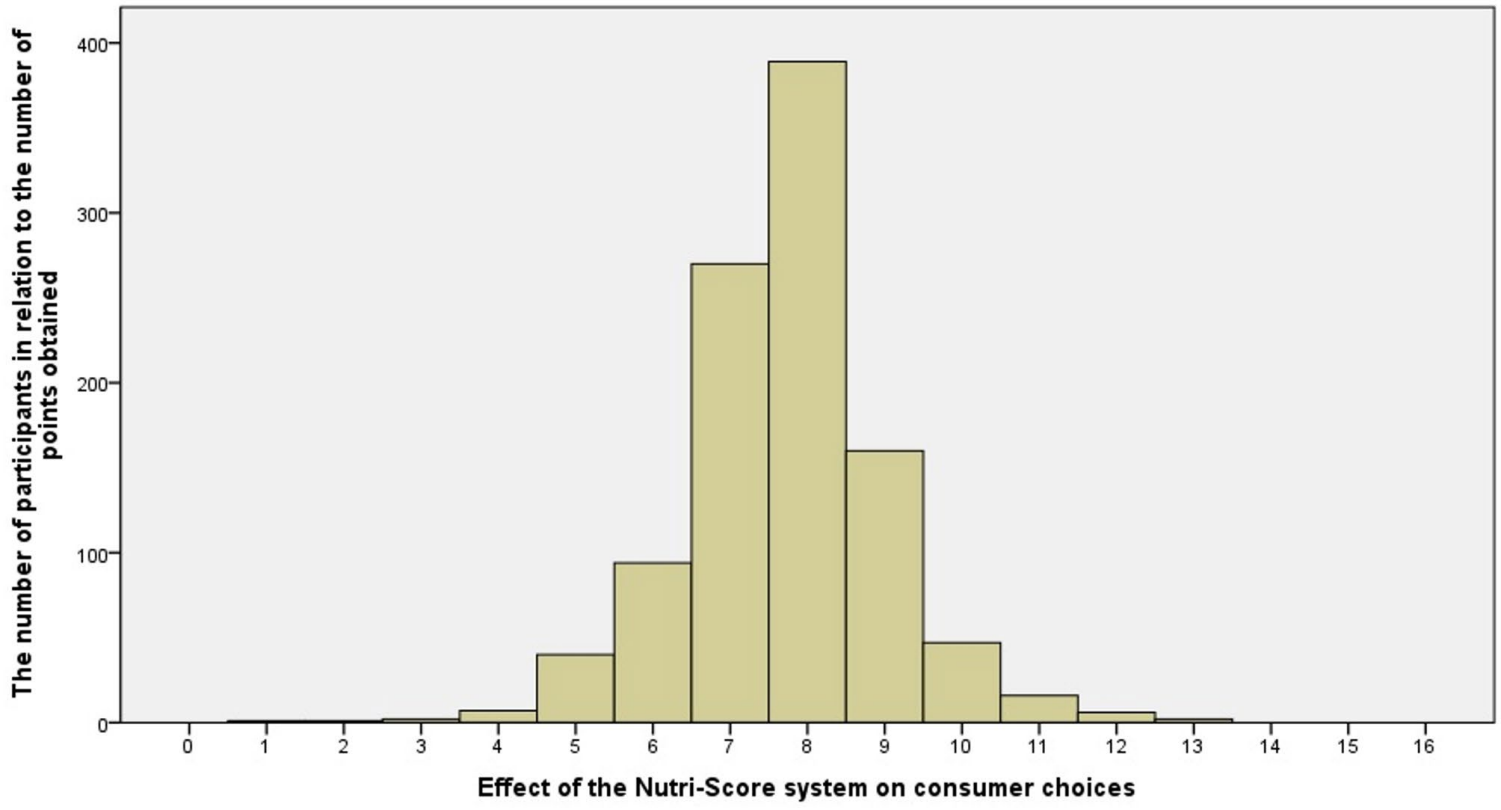
Effect of the Nutri-Score system on consumer choices.

This study examined the final impact of the Nutri-Score labelling system on enhancing consumer opinion during the trial period. The results, presented in Histogram 1 (Fig. 1), highlighted a non-normal distribution of data, as confirmed by the Kolmogorov–Smirnov test (z = 0.192; *p* < 0.001), indicating significant deviations from the expected outcomes under normal distribution assumptions.

Histogram analysis revealed a clear uplift of the left portion of the graph compared to the right, centred around a median score of 8 points, predefined as the neutral point of reference. This skew suggests that a substantial number of participants scored below the neutral point, with an average score across participants of 7.71 (SD = 1.30), signifying a tendency towards less favourable opinion on the healthiness of the productwhen exposed to Nutri-Score labels.

This distribution pattern suggests that while some consumers were positively influenced by Nutri-Score labels to make healthier choices, a significant portion of the sample did not achieve scores that would indicate a strong positive shift in behaviour due to label visibility.

### Correlation between knowledge scores and consumer opinion about healthiness of the food product under Nutri-Score labelling

The analysis, leveraging Spearman’s correlation test, demonstrated a statistically significant correlation between Nutritional Knowledge Test scores and consumer opinions about products when Nutri-Score labels were obscured (rho = 0.216, *p* < 0.001) and visible (rho = 0.164, *p* < 0.001). This indicates that higher knowledge scores were associated with more health-conscious food selection under both conditions. However, no significant correlation was found between the overall effect of the Nutri-Score system on consumer choices and the total points achieved by the participants in the Nutritional Knowledge Test (*p* = 0.10, rho = −0.051). This suggests that, while individual knowledge may influence opinion about specific products, it does not necessarily predict the overall impact of the Nutri-Score labelling on food selection patterns across the study sample. In addition, there was a negative correlation between knowledge of the Nutri-Score system, as demonstrated by a test of knowledge of the system, and the final effect of this system on consumer opinion on the healthiness of the product (rho = −0.082; *p* = 0.008, Spearman’s test).

## Discussion

The main objective of this study was to examine the effect of the Nutri-Score system on consumer opinion regarding food healthiness in the context of nutritional value, with the participation of a representative group of Polish consumers. To the best of our knowledge, this is the first study to evaluate Nutri-Score in this context on Polish consumers. In addition, an unquestionable advantage of this study is that it was conducted using the latest version of the Nutri-Score system algorithm, for which there are few studies on an international scale. This represents a unique contribution of our work to the current state of knowledge and discussion on a common, unified FOPL system across the European Union, which the European Food Safety Authority (EFSA) believes needs to be introduced^25^.

The first important issue in the results is whether the Nutri-Score system influences consumer opinion when choosing food products. As the analysis showed, in the majority (six out of eight pairs) of cases, the Nutri-Score system did not influence consumer choices in a statistically significant way. Only two pairs showed statistically significant differences (*p* < 0.001, McNemar’s chi-square test in both cases). Many factors influence consumer opinion when selecting a food product, such as quality, freshness, price, taste, healthiness, consumer demographic characteristics (including age, gender, and education), and product availability^26,27^, some of which may affect the effectiveness of FOPL, including the Nutri-Score system^28^. Therefore, it is difficult to unequivocally assess the reasons for the lack of change in decisions. One likely reason is that the individual study participants were persuaded to choose a healthier product with both obscured and unveiled Nutri-Score system scores. A second, more likely reason is that there were no significant differences in Nutri-Score scores between the individual paired products. A clear difference in rating (colour change and ≥ 2 grade change in rating system) occurred in the last two product pairs, which showed a statistically significant change in product choice. As in the remaining product pairs, the difference in rating with the Nutri-Score system was small (same rating in both products or a change of 1 degree with minimal colour change of dark green to green), consumers mostly stayed with their, more or less, correct choice. This suggests that when comparing a pair of products with a similar Nutri-Score, the system has little or no impact on consumers’ assessment of product health. However, this interpretation should be approached with caution, as the limited number of product pairs and the specific context in which choices were made (e.g., experimental setting, limited product categories) may not fully reflect the complexity of real-world consumer behaviour. The influence of this system on consumers’ assessment of product health and opinion on the healthiness of the product is visible only in the case of a larger and more visible spread. This finding is supported by previous studies. According to these studies, colour and preference are seen as factors influencing consumer choices^29,30^. In contrast, the colours used in FOPL can enhance the perception of the healthiness of products^28^. The lack of a clear change in the colour of the FOPL may therefore give consumers the impression of similar or the same healthiness of the product.

However, these findings raise some concerns. There is a possibility that if two products, one more and the other less beneficial to health, are given similar scores, this could be confusing for consumers. In addition, the system could be used to justify one’s food choices according to one’s own tastes and criteria. These findings highlight the need to enhance the discriminatory power of FOPL systems, potentially by refining the Nutri-Score algorithm to better differentiate between products of varying nutritional quality (for example, by extending the algorithm to include as many components as possible), so that it effectively distinguishes between two products of different healthiness and does not give the same rating, as was the case in this study.

The second and more relevant question is whether the lack of the aforementioned change in the six pairs of products, and the existing change in the two pairs of products, are positive or negative events. This is because the pairs compared did not contain identical products; therefore, one had more positive characteristics than the other. In terms of the regularity of any changes in decisions, a higher percentage of consumers changed their product choice to a less favourable one for yoghurt (9.57% vs. 7.15%), bread (12.17% vs. 11.11%), and pasta (11.21% vs. 8.60%), while a higher percentage of study participants changed their choice to a more favourable one for beverage (8.60% vs. 8.02%), breakfast cereal (9.18% vs. 7.54%), and herring (8.60% vs. 8.12%). However, these changes were not statistically significant. Statistically significant (*p* < 0.001, McNemar’s test) changes were observed for two pairs of products: fish and a comparison between sardines and salmon (18.74% vs. 6.09%), and ham and a comparison between regular pork ham and Serano ham (25.22% vs. 12.95%). As mentioned, these were pairs of products where the change in rating according to the Nutri-Score system was ≥ 2 rating categories. Consumers, probably following the rating, decided to change to a product with a better rating according to the Nutri-Score system, since the decision change in these two cases was from a worse to a better Nutri-Score: from category E to category B in the case of fish choices and from category E to category C in the case of hams. This further suggests a mechanism for how Nutri-Score works: since for the same or comparable ratings and colours the system does not influence consumer decisions, and for larger differences, consumers make unfavourable selections, this suggests that the Nutri-Score system’s ability to influence diet quality may depend on the magnitude of the rating differences between products and is only based on a simple understanding of colours, where red is associated with a ‘stop’ signal and green with an ‘eat’ signal, and yellow is a neutral point between two sides. This mechanism was suggested in a previous study^31^. An alternative explanation may relate to the broader decision-making context, including consumers’ prior knowledge, brand loyalty, or habitual purchase behaviour, which can modulate the influence of front-of-pack labels regardless of colour coding.

It is worth highlighting that in both cases where a statistically significant change was shown, the decision was less favourable because, when the Nutri-Score was revealed, the less favourable product was more often indicated as healthier than when the score was not visible to the consumer. This was due to the fact that the better products received worse ratings in both cases. When comparing salmon with sardine fillets, hot-smoked salmon fillets were identified as the more favourable product. The product is lightly processed for preservation. It has a high raw material content (up to 98%) and is a much better source of omega-3 polyunsaturated fatty acids than sardine fillets in oil. In the case of hams, on the other hand, Serrano ham was chosen as the more favourable product. This is a regional product with unique sensory characteristics. Despite its higher energy value and almost twice the protein content, this ham is occasionally consumed in small quantities. At this stage, it is worth pointing out that the Nutri-Score is a system that bases its assessment not on the usual consumed portion of a product, but on the nutritional value per 100 g or ml. This unique production method ensures superior taste and quality. The product in the form of tinned pork ham is consumed in much larger quantities; therefore, despite its lower energy value, it provides more unfavourable ingredients such as saturated fatty acids, additives resulting from the processing of the product (e.g. preservative sodium nitrite), or more energy. However, the unfavourable change may have been due to apparent changes in assessment by the Nutri-Score system. Participants in the study may have bypassed the quality criteria for Serrano ham given that this would have required knowledge of the quality parameters in assessing hams and their importance.

In the case of small rating differences, the results suggest that the Nutri-Score system had a negligible impact on consumer opinion on the healthiness of the product, whereas in the case of products where rating differences were apparent and the observed changes in opinion of the product were considered less favourable, this can be perceived as a confusing impact of the Nutri-Score system for the average consumer. This may reflect structural limitations in the current version of the algorithm and indicates a need for its ongoing refinement. Although the system is constantly improving, it still has many imperfections, as shown. Nevertheless, it should be acknowledged that even simplified nutrient profiling systems may serve as valuable tools in guiding public health behaviour when appropriately contextualized and combined with broader nutrition education strategies. These flaws, such as the lack of consideration of fatty acid content, packaging and portion size, degree of processing of the product, and its regional origin, have been demonstrated in previous studies. Włodarek and Dobrowolski (2022) showed that the Nutri-Score system does not account for factors such as package size, vitamin/mineral content, degree of processing, or fatty acid profile^31^. Romero Ferreiro et al. (2021) also indicated that the Nutri-Score system does not consider the degree of food processing^32^. The 75 Polish experts participating in the nationwide survey also indicated that the Nutri-Score system does not consider a product’s degree of processing or total nutritional value^33^. A Norwegian study by Paulsen et al. (2024) also indicated problems in assessing meat, fish, and high-fat or highly processed products, among others^34^. Although some of the flaws mentioned in numerous studies have been corrected or eliminated^35^, some errors still exist (e.g. not considering the degree of food processing, not including vitamins and minerals or selected fatty acids in the algorithm), as this study highlights.

Finally, a third important aspect is what effect the Nutri-Score system ultimately has on consumer opinion on the healthiness of the product. An analysis of the graph of the effect of the Nutri-Score system shows that although the effect was neutral on a significant percentage, the predominant effect was negative, with the system itself producing more adverse changes in food choices than positive changes. This effect is directly attributable to the changes in the opinions taken by consumers on the healthiness of the products on a pair-by-pair basis, with the Nutri-Score label obscured and then exposed. Given that most were negative (five product pairs) and a few were positive (three product pairs), the final effect was negative. This interpretation should, however, be contextualised by the limited number of product comparisons and the controlled conditions under which the data were collected. It is possible that consumer behaviour in real-life retail environments, where multiple cues are present, may yield different outcomes.

Current studies have indicated both beneficial^36–38^ and unfavourable dietary choices^39–41^ using the Nutri-Score system under product selection conditions, depending on the research model. A number of studies have also shown a more favourable evaluation by the Nutri-Score system of products with a health-promoting nutritional profile and indicated that it helps consumers make healthier dietary choices^20,21,38,42,43^. However, this studies were conducted prior to the latest system update. The updated version of the system was the subject of a study by Sarda et al. (2024), who found that the updated algorithm evaluates products in better agreement with French healthy eating recommendations than the previous version and therefore shows better performance in making health-promoting dietary choices^35^. However, this study was conducted by the authors of the system.

It is worth noting the negative correlation between Nutri-Score’s effect on consumer opinion on the healthiness of the product and knowledge of this system. Knowledge of the Nutri-Score system was collected using a self-administered questionnaire based on publicly available information used to promote the system in retail chains and grocery shops. Therefore, greater knowledge of the system may have been obtained from promotional materials convincing people of the high informational value and promotion of health-promoting foods by this system, while not informing them of the limitations of this system. This raises questions about the comprehensiveness and balance of public information campaigns and whether they sufficiently equip consumers to critically evaluate front-of-pack nutrition labelling. In the present study, the negative impact of the Nutri-Score on consumer opinion on the healthiness of the product was precisely due to the limitations and imperfections of the algorithm on selected issues. However, more favourable food choices were shown among study participants with higher nutritional knowledge. These results are in agreement with those obtained by Hemar-Nicolas et al. (2024), who demonstrated an inadequate understanding of both the Nutri-Score algorithm and the influence of nutritional knowledge on the correct application of labels^44^. Lack of adequate nutritional knowledge with mass communication of the advantages of promotional material about the scheme can cause consumers to misconceive of the infallibility of the system and lead to unfavourable nutritional choices, as was the case in this study.

### Limitations

The present study, apart from its robust methodology and being carried out in an extremely precise and detailed manner, is not without flaws. First, it was conducted on a limited number of products in a specific context. Although eight product pairs were used, they were mostly from different food groups which differed in their assessments using the Nutri-Score system. Different results could have been obtained if other product pairs were used. However, it should be pointed out that other experiments were often conducted using a smaller number of food products. In addition, some flaws in the performance of the algorithm behind the Nutri-Score are highlighted. If these pairs have flaws, they will likely also exist in other products. At a time of discussion about a common mandatory FOPL system across the European Union, there is a need for a system with unquestionable efficacy and a broad approach to the product, which will ensure that it works and allow consumers to make healthier food selections.

The second limitation of the study is the form of question formulation. The research protocol used caused products to be presented first with the Nutri-Score label hidden and then with the Nutri-Score label exposed. On this basis, study participants may have unconsciously concluded that they should rely solely on the Nutri-Score system when assessing products, instead of the other food quality attributes that the study authors presented on the packaging. It should be noted though, that a different formulation of the questions could in turn have diverted attention away from Nutri-Score labelling. However, the authors’ intention was to induce only one variable that would best capture the effect of the presence of Nutri-Score labelling on packaging.

Another limitation is the design of this study. It is not an experimental study, and although some relationships may be observed, their directionality or causative nature remains unclear. However, although this study does not establish causation, it raises questions about whether the current design of the algorithm can be used in its present form. Therefore, the present results despite their limitations, may point to potential directions for changes in the algorithm. However, other studies are needed, especially focusing on the sale of Nutri-Score-labelled products in real-world shop settings, in order to clearly determine the impact of the label on food product preferences and consumer choices.

## Conclusions

In conclusion, this study shows that the Nutri-Score system has a limiting effect on improving consumer opinion on the healthiness of the product in terms of choosing healthier food products. In most cases, the observed impact was negligible, or induced selection changes that could be considered negative. In contrast, a relationship between making more favourable nutritional choices and nutritional knowledge was demonstrated regardless of the visibility of the Nutri-Score label, suggesting that proper nutritional education is still a key element in improving consumers’ nutritional choices. Further studies are needed to determine whether this association reflects a causal mechanism.

## Methods

### Design

This cross-sectional study was conducted in March and April 2024 using the Computer-Assisted Web Interview (CAWI) technique.

### Setting

This study was conducted nationally across Poland, using an online research panel to collect data.

### Sample selection

The sample was representative and was selected through a quota system from respondents who were registered and invited to participate in a national research panel. The selection was randomised within each quota until the sample was fully realised, based on the distribution of sociodemographic characteristics (sex, age, education level, and place of residence including town size and province) as per data from the National Census of Population and Housing 2021^45^. The sample selection criteria determined the inclusion of each respondent, and identification was based on the responses to demographic questions in the survey.

### Sample size

Initially, 1067 respondents participated, representative of the adult Polish population, with a confidence level of 95% and a margin of error of 3%. After the study, 32 participants were excluded because of their specialised knowledge of health sciences, nutrition, and dietetics, leaving 1035 participants for data analysis.

### Participants

Participants were adult residents of Poland who declared that they had purchased food items from retail chains within the last month. The exclusion criteria included individuals under the age of 18 years, those with cognitive impairments, those who had not made retail purchases within the previous month, and those who were unwilling or unable to provide informed consent.

### Instrument and measurement

In this study, we used a survey method using a questionnaire developed collaboratively by experts in medicine, dietetics, human nutrition, legal aspects of food labelling, psychology, social research methodology, and statistics. The development process began with an extensive review of the scientific literature on food labelling to ensure the contextual relevance of the questionnaire. The experts compiled a structured list of topics and created a questionnaire bank. This bank underwent critical review and refinement before the preliminary version was produced. This version was further enhanced through reviews by external experts and a pilot study.

The instrument used was a comprehensive questionnaire developed to evaluate both the sociodemographic background and nutritional knowledge of the participants, their knowledge of Nutri-Score labelling, and their decision-making in food choices influenced by nutritional labelling.

The initial part of the questionnaire consisted of metric questions that gathered data on sex, age, education, place of residence, professional and material situation, household size, presence of children under the age of 13, chronic diseases, and adherence to special diets. Data collection was pivotal for understanding the socioeconomic context of the respondents and ensuring a representative sample of the Polish population.

Subsequently, the questionnaire assessed participants’ nutritional knowledge and beliefs about healthy eating using ten questions derived from the KomPAN Questionnaire^46,47^. This section, the Nutritional Knowledge Test, aimed to evaluate the respondents’ understanding of the key aspects of a proper diet and common nutritional myths. Participants were asked to identify whether the statements presented to them were true or false, scoring one point for each correct answer, with a maximum of 10 points achievable. The detailed contents of these questions are presented in Table 5.

**Table 5:** Nutritional knowledge test from the KomPAN Questionnaire^46^.

No	Statement	Correct answer
1	It is sufficient to consume cereal products once a day.	False
2	Fruits and/or vegetables should be consumed with every meal.	True
3	High salt intake protects against hypertension.	False
4	Reducing fatty foods in the diet helps prevent cardiovascular diseases.	True
5	Bio-yogurts contain beneficial intestinal bacteria.	True
6	Oil and olive oil contain a lot of cholesterol.	False
7	Wholemeal bread contains more fiber than white bread.	True
8	Fruits and vegetables are a source of “empty calories.”	False
9	Yellow cheese is a better source of calcium than cottage cheese.	True
10	Protein should be the main source of energy in a proper diet.	False

Following the nutrition knowledge assessment, participants were asked about their knowledge of Nutri-Score labelling, which was intended to assess their understanding of the principles and scope of labelling in the context of evaluating food products, thus indicating whether respondents were able to interpret the information indicated by the Nutri-Score system correctly. The questions included only the basic knowledge promoted in the social campaigns of the algorithm’s creators and grocery stores. The questions and their correct answers are listed in Table 6. Possible answer options were “Yes”, “No” and “Don’t know”. One point was given for a correct answer, while no points were given for an incorrect answer or a “don’t know” answer. Respondents could obtain a maximum of 7 points for all answers.

**Table 6:** Knowledge of the Nutri-Score labelling system test.

No	Question	Correct answer
1	Does Nutri-Score use a grading/colour scale (red to green) to assess the nutritional value of foods?	Yes
2	Does Nutri-Score use a graded numerical scale (1 to 5) to assess the nutritional value of food?	No
3	Does Nutri-Score use a graded alphabetical scale (E to A) to assess the nutritional value of foods?	Yes
4	Can Nutri-Score be used to compare the nutritional value between two products belonging to the same category (e.g. cheese with another cheese or juice with another juice)?	Yes
5	Can Nutri-Score be used to compare the nutritional value between two products belonging to different categories (e.g. cheese with juice or bread with cold cuts)?	No
6	Does Nutri-Score have another, separate designation for dangerous products?	No
7	Does the Nutri-Score system rate food products differently, i.e. cheeses, beverages, baked goods, and cured meats?	Yes

**Table 7:** Comparative evaluation of paired food products: Nutri-Score labelling 795 and brief characteristics.

Product group	Product	Nutri-Score*	Product characteristics **
Yogurts	**Creamy yoghurt with strawberries**	**C** **Yellow**	**Skimmed milk, fruit filling (strawberries, sugar, carrageenan, xanthan gum), cream, natural flavouring. Contains vitamin C. Lower calories.**
	Creamy yoghurt with chocolate-covered flakes	C Yellow	Skimmed milk, cream, chocolate flakes (corn grits, sugar, cocoa fat). Contains added sugar. Higher calories.
Bread	**Multi-grain bread. Whole-grain rye bread, sliced**	**A** **Dark Green**	**Whole-grain rye middlings, water, barley flakes, oat flakes, flaxseed. Rich in fibre.**
	Whole-grain toast bread	B Green	Whole-grain wheat flour, wheat flour, wheat sourdough, rapeseed oil. Less fibre, contains refined flour.
Pasta	**Penne rigate wholegrain pasta**	**A** **Dark Green**	**Wheat flour from durum wheat. Higher fibre content, more beneficial effect on glycaemia.**
	Penne rigate pasta	A Dark Green	Wheat flour from durum wheat. Less fibre, worse effect on glycaemia.
Beverages	**Apple carrot raspberry Juice**	**C** **Yellow**	**Apple juice, carrot and raspberry puree, vitamin C. Source of vitamins A and C, contains naturally occurring sugars and polyphenols.**
	Tea drink	C Yellow	Water, tea extract, peach juice from concentrate, sweetener. No nutritional value.
Breakfast cereals	**Muesli** **Fruit, flaxseed, pumpkin seeds**	**C** **Yellow**	**Whole-grain barley flakes, raisins, oat flakes, banana chips, candied mangoes, flaxseed. Source of fibre and omega-3.**
	Chocolate-flavoured cereal in the shape of balls	C Yellow	Wholegrain wheat flour, maize groats, glucose syrup, cocoa, less fibre.
Herrings	**Herring fillets in tomato sauce with paprika**	**C** **Yellow**	**Herring fillets, tomato sauce, peppers, onions. Rich in omega-3 fatty acids, antioxidants from tomatoes.**
	Herring fillets in vegetable oil	C Yellow	Herring fillets, rapeseed oil. Less favourable type of fat, no vegetable addition.
Fish	**Hot smoked Atlantic salmon - bellies with peel**	**E** **Dark Orange**	**Atlantic salmon, minimal processing. High in omega-3 fatty acids, beneficial for the cardiovascular system.**
	Fillets of sardines in extra virgin olive oil	B Green	Sardines, extra virgin olive oil. Less omega-3 fatty acids, more processed.
Hams	**Serrano pork ham, dry-cured, matured**	**E** **Dark Orange**	**Pork ham, minimal preservatives, high protein content, regional product with a unique flavour, high sensory appeal.**
	Pork ham	C Yellow	More preservatives (nitrates, nitrites), relatively low protein content, more processed.

* Food quality rating system updated in 2024, ranging from A (most nutritionally favourable option) to E (least nutritionally favourable option).

** Bold: Indicates the product deemed more nutritionally beneficial by a panel of experts compared to the other product in the pair.

The second component of the survey involved presenting participants with eight pairs of food products from various categories, including yoghurts, bread, pastas, beverages, cereals, fish, and ham. Detailed information on the composition and nutritional value of these products was provided. Each product was assigned a Nutri-Score category based on the latest version of the effective algorithm from 2024^14^. Participants compared these products based on their nutritional value and chose the product from each pair that they believed was better aligned with the healthy diet recommendations. This evaluation process was conducted twice, initially without and then with the Nutri-Score labels displayed, to determine the influence of this labelling on consumer choices. Products were shown to consumers in random order to minimise the confounding factor of remembering previously given answers and to ensure greater objectivity in evaluating different forms of labelling. All products presented to the respondents, together with their group assignments, Nutri-Score ratings, and brief characteristics, are presented in Table 7. In the supplementray materials in Table A, full information is presented on the packaging (translated into English - in the original, the content was presented in Polish) presented to study participants, as well as a miniature of the packaging. Table B presents a full-size image of the packaging presented to study participants.

**Table 8:** Scoring system for evaluating the impact of Nutri-Score visibility on consumer choices.

Score	Condition	Explanation
**0 points**	Participant chose a more beneficial product without seeing the Nutri-Score, switched to a less beneficial product when Nutri-Score was visible.	Suggests a negative influence of Nutri-Score, where its visibility led to less healthy choices.
**1 point**	Participant chose the same product both with and without Nutri-Score visibility.	Indicates consistency in decision-making, showing no influence from Nutri-Score labelling.
**2 points**	Participant chose a less beneficial product without the Nutri-Score, switched to a more beneficial product when Nutri-Score was visible.	Indicates a positive influence of Nutri-Score, where its visibility encouraged healthier choices.

A scoring system was implemented to assess the impact of the Nutri-Score system on consumer decisions (Table 8). This scoring system aimed to evaluate the effectiveness of the Nutri-Score in swaying consumer choices towards healthier options. The results were plotted on a continuous scale from 0 to 16 points, where:

- A score close to 0 indicated a predominance of negative influences by the Nutri-Score, leading participants away from healthier choices.
- A score close to 16 indicated a significant positive impact, steering participants towards healthier options.
- The midpoint of 8 points suggested a neutral impact of the Nutri-Score system on consumer choices, either by not influencing consumer decisions or by an equivalent amount of positive and negative impacts of the system on individual products.

This method provides a clear quantification of how Nutri-Score labelling affects consumer choices, allowing for a straightforward interpretation of its impact in promoting healthier eating habits.

### Statistical methods

Statistical analyses were performed using the SPSS software (version 27.0; IBM Corp.). Normality of the data distribution was assessed using the Kolmogorov–Smirnov test.

For each product pair, a 2 × 2 contingency table was constructed from participants’ pre- and post-label choices. McNemar’s exact test assessed whether the proportion switching changed significantly when the Nutri-Score became visible. To quantify the direction and magnitude of that change, we computed an odds ratio (OR) defined as c/b, where.

-  b = number of discordant cases that switched toward the expert-defined healthier product after seeing the label,
-  c = number of discordant cases that switched away from the healthier product.

Thus OR > 1 indicates an increased likelihood of less favourable choices attributable to label visibility, whereas OR < 1 indicates a net shift toward the healthier option; OR = 1 implies no directional change. Exact mid-P 95% confidence intervals for each OR were obtained with the mcnemar.exact function from the R package Exact. The relationship between the impact of the Nutri-Score system on consumer choices and nutritional knowledge, as well as knowledge of the Nutri-Score system, was examined using Spearman’s rank correlation analysis. A significance level of α = 0.05 was set for all tests. 

## Supplementary Information

Below is the link to the electronic supplementary material.

Supplementary Material 1

Supplementary Material 2

## Data Availability

The datasets generated during and/or analysed during the current study are available from the corresponding author on reasonable request.
