# Supplementary material for: Assessment of the influence of Nutri-Score on Polish consumer Choices - Insights from the nationwide, Cross-sectional study
Source: Sci Rep. 2025 Aug 11;15:29422. doi: 10.1038/s41598-025-14033-9 (PMC12339969; doi:10.1038/s41598-025-14033-9)
Supplement: Supplementary file 1 — Supplementary Material 1 [file 41598_2025_14033_MOESM1_ESM.docx]

| **Product** | **Pair** | **Name** | **Composition (translation)** | **Nutri-Score** | **Miniature picture** |
| --- | --- | --- | --- | --- | --- |
| 1 | 1 | Creamy yoghurt with strawberries | **Ingredients**: skimmed milk, fruit filling (strawberries 13.7%, sugar, modified starch, thickeners: carrageenan, xanthan gum, black carrot juice concentrate, acidity regulators: sodium citrate, citric acid; natural flavour), cream, sugar, live yoghurt bacteria cultures, skimmed milk powder.  **Nutritional Facts** per 100 g of product: energy 496 kJ/118 kcal; fat 5.3 g, including saturated fat 3.4 g; carbohydrates 15.2 g, including sugars 14.8 g; protein 2.3 g; salt 0.09 g. | C  Yellow | 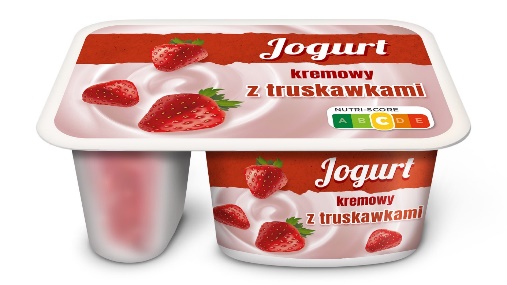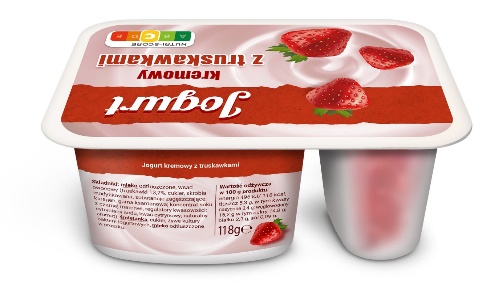 |
| 2 |  | Creamy yoghurt with chocolate-covered flakes | **Ingredients**: yoghurt: skimmed milk, cream, sugar, skimmed milk powder, live yoghurt bacteria cultures; chocolate-covered (6.9%) cornflakes (11%): 32% corn grits, sugar, cocoa fat, whole milk powder, cocoa pulp, salt, emulsifier (lecithins (from soya and sunflower)), barley malt extract, natural vanilla flavouring.  **Nutrition Facts** per 100 g of product: energy 637 kJ/152 kcal; fat 7.9 g, including saturated fat 5.0 g; carbohydrates 16.8 g, including sugars 13.7 g; protein 3.4 g, salt 0.19 g | C Yellow | 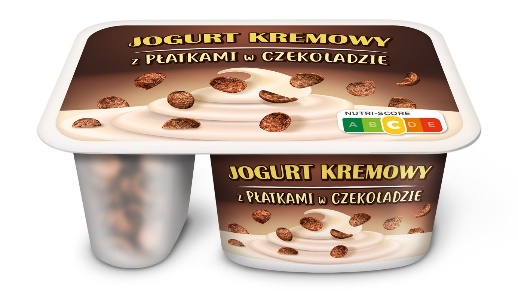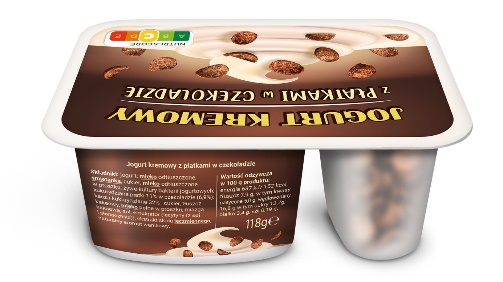 |
| 3 | 2 | Multi-grain bread. Whole-grain rye bread, sliced | **Ingredients**: Whole-grain rye middlings (52%), water, barley flakes (3%), oat flakes (3%), flaxseed (3%), salt, yeast  **Nutrition Facts** per 100 g: Energy value 846 kJ/201 kcal, fat 2.4 g, of which including saturated fat 0.4 g, carbohydrates 34.7 g of which sugars 2.2 g, fibre 9.4 g, protein 5.5 g, salt 1.1 g | A  Dark Green | 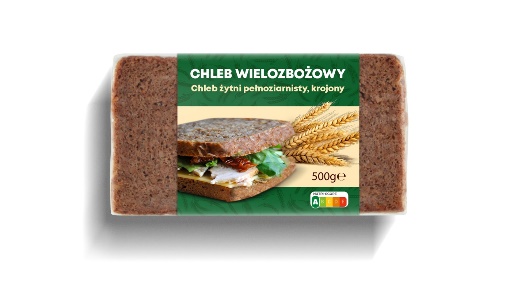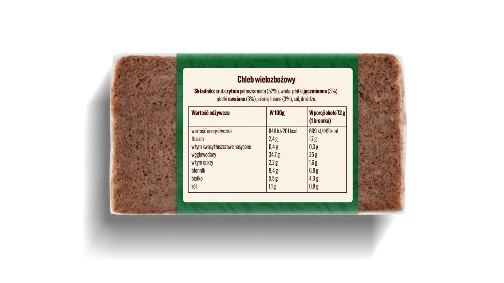 |
| 4 |  | Whole-grain toast bread | **Ingredients**: Whole wheat flour 35.3%, wheat flour 30.2%, water, wheat sourdough 5% (wheat flour, water), yeast, salt, canola oil, spirit vinegar, sugar, soy flour  **Nutrition Facts** per 100 g: Energy value 1010 kj/239 kcal, fat 2.5 g, of which saturated fat 0.4 g, carbohydrates 43 g, of which sugars 2.8 g, fibre 5.9 g, protein 8.2 g, salt 1.2 g | B  Green | 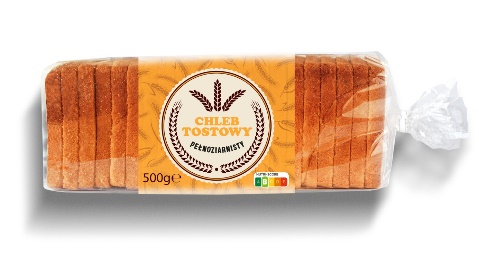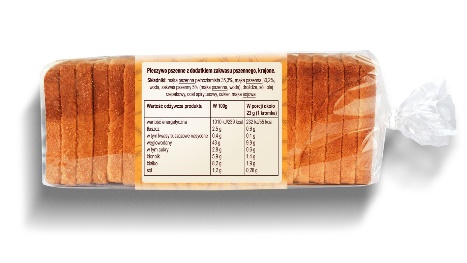 |
| 5 | 3 | Penne rigate wholegrain pasta | **Ingredients**: Hard wheat (durum) flour.  **Nutrition Facts** per 100g: Energy value 1502 kj/355 kcal, fat 2.1 g, of which saturated fat 0.4 g, carbohydrates 67.7 g, of which sugars 2.6 g, fibre 7.0 g, protein 12.8 g, salt 0.150 g. | A  Dark Green | 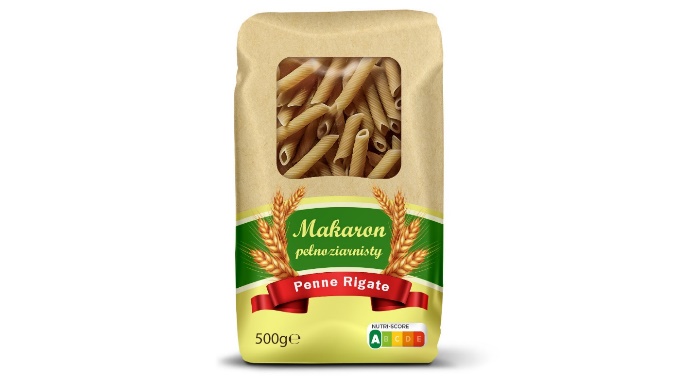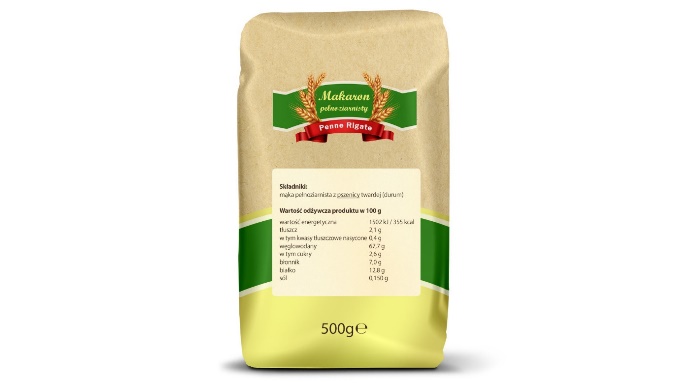 |
| 6 |  | Penne rigate pasta | **Ingredients**: Hard wheat (durum) flour.  **Nutrition Facts** per 100g: Energy value 1478 kj/349 kcal, fat 1.5 g, of which of which saturated fat 0.3 g, carbohydrates 70 g, of which sugars 2.9 g, fibre 3.6 g, protein 12 g, salt 0.03 g | A  Dark Green | 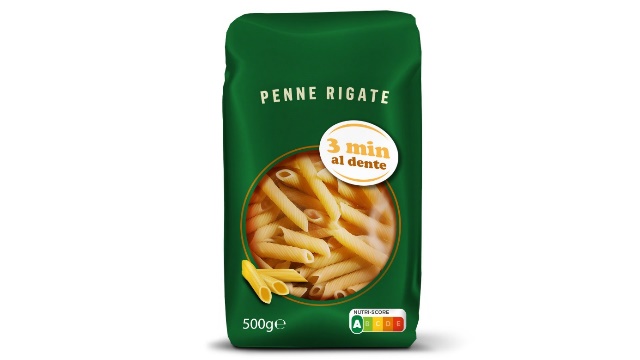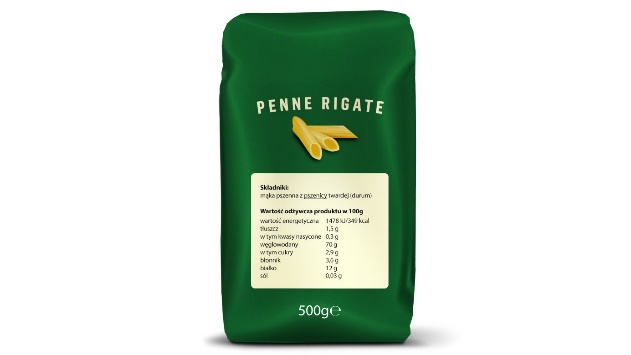 |
| 7 | 4 | Apple carrot raspberry Juice | **Ingredients**: Apple juice from concentrated juice (57%), purees of: carrots (37%), raspberries (4%) and apples (2%), vitamin C, flavouring  **Nutrition Facts** per 100 ml: Energy 179 kJ/42kcal, fat 0.2g, of which saturated fat 0.02g, carbohydrates 9.1g, of which sugars 9.0g, fibre 1.4g, protein 0.4g, salt 0.06g, vitamin C 24mg (30% of GDA), vitamin A (50% of GDA). The salt content is solely due to the presence of naturally occurring sodium. This sugar comes exclusively from fruit and carrots. | C Yellow | 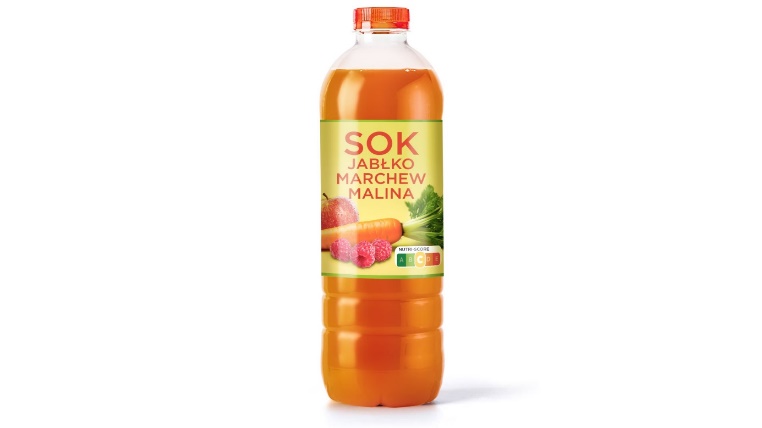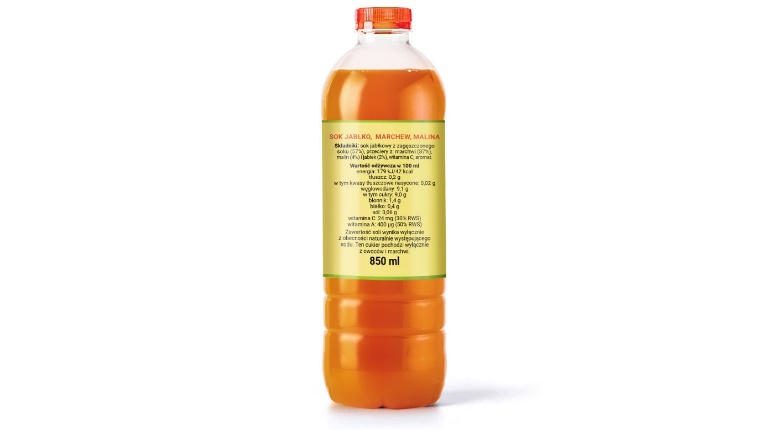 |
| 8 |  | Tea drink | **Ingredients**: Water, acidity regulator: citric acid, tea extract 0.12%, peach juice from concentrate 0.1%, flavouring, antioxidant: ascorbic acid, sweeteners: acesulfame-K and sucralose.  **Nutrition Facts** per 100 ml: Energy 6 kj/1 kcal, Fat 0.0 g, of which saturated fat 0.0 g, Carbohydrates 0.0, of which sugars 0.0 g, Fibre 0.0 g, Protein 0.0 g, Salt 0.03 g | C Yellow | 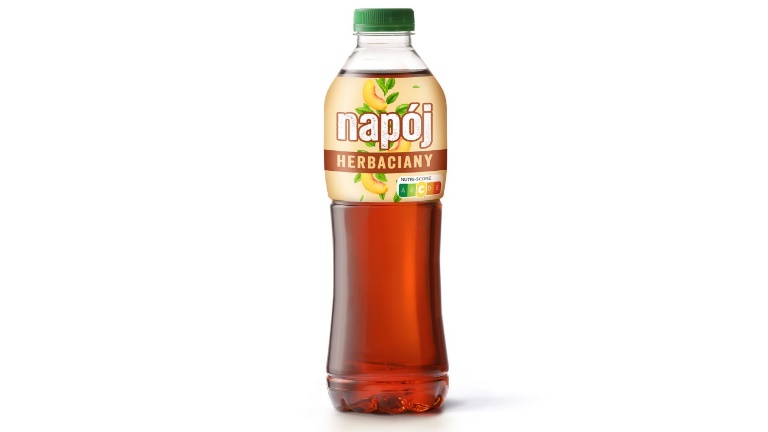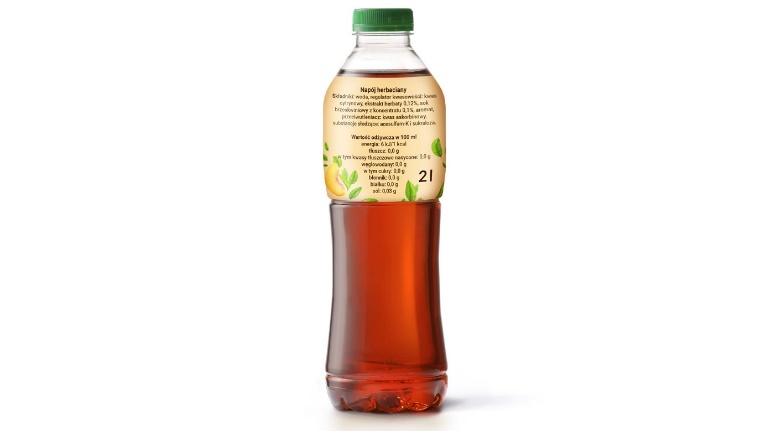 |
| 9 | 5 | Muesli  Fruit, flaxseed, pumpkin seeds | **Ingredients**: 25.5% whole grain barley flakes, 25% craisins (99.5% craisins, cotton and/or sunflower oil), 15.5% whole grain oat flakes, 7% banana chips (58% bananas, coconut oil, cane sugar, banana flavouring), 5% candied cubed pineapple (53% pineapple, sugar, acidity regulator: citric acid), 5% candied cut mango (71% mango, sugar, acidity regulator: citric acid; preservative: sodium pyrosulphite), 4.5% flaxseed, 4% whole wheat flakes, 3.25% pumpkin seeds, 2% freeze-dried plums pieces, 2% dried peaches cubed (96% peaches, 4% rice flour), 1.25% dried apricots cut (97% apricots, rice flour, preservative: sulphur dioxide).  **Nutrition Facts** per 100g: Energy value 1638 kj/389 kcal, fat 8.9 g, of which saturated fat 2.9 g, carbohydrates 65 g, of which sugars 29.5 g, fibre 7.6 g, protein 8.4 g, salt 0.12 g | C Yellow | 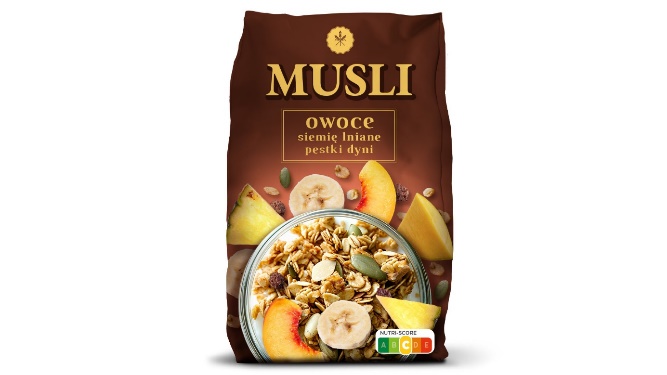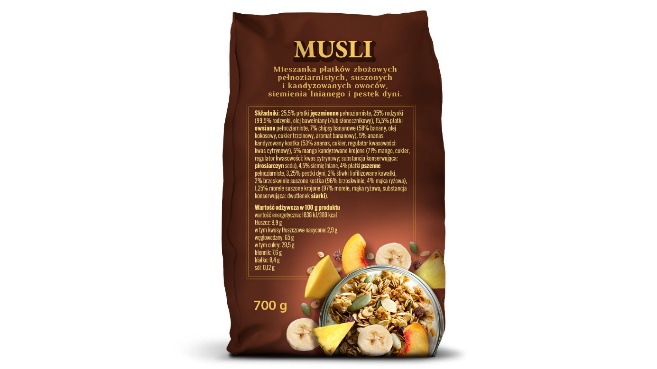 |
| 10 |  | Chocolate-flavoured cereal in the shape of balls | **Ingredients**: 52% whole wheat flour, 14.5% sugar, maize groats, glucose syrup, 5.7% reduced-fat cocoa powder, salt, natural flavours, enrichments: vitamin E, thiamin, riboflavin, niacin, vitamin B6, folic acid, pantothenic acid, calcium, iron.  **Nutrition Facts** per 100g: Energy value 1537 kj/363 kcal, fat 2.2 g, including saturated fat 0.6 g, carbohydrates 72.8 g, including sugars 23.9 g, fibre 7.7 g, protein 9.2 g, salt 0.45 g, vitamin E 12 mg (100%), thiamin 1, 1 mg(100%), riboflavin 1.4 mg (100%), niacin 16 mg (100%), vitamin B6 1.4 mg (100%), folic acid 200 mcg (100%), pantothenic acid 6 mg (100%), calcium 400 mg (50%), iron 7 mg (50%) | C Yellow | 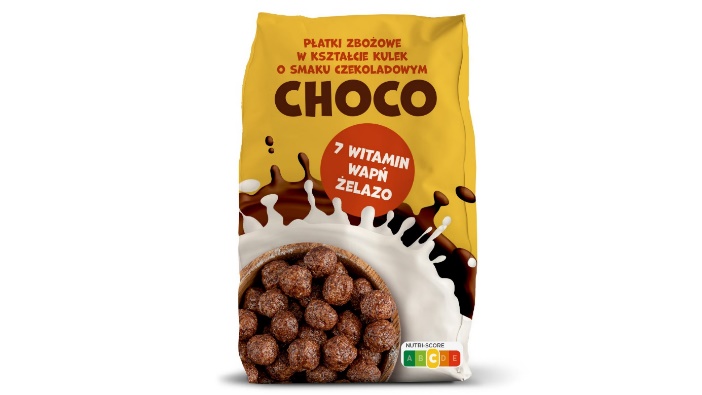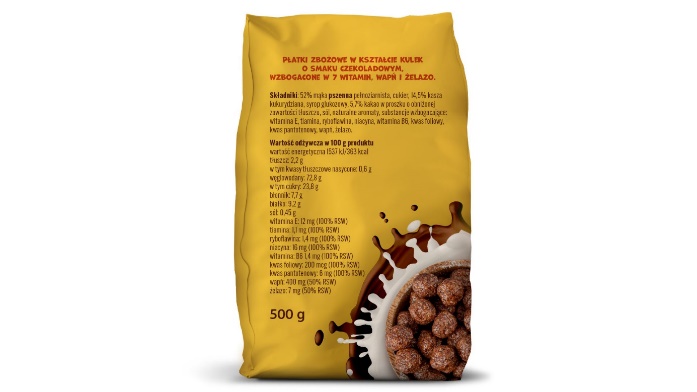 |
| 11 | 6 | Herring fillets in tomato sauce with paprika | **Ingredients**: Fish - herring fillets 60%; tomato sauce 35.5%: water, tomato concentrate 20.48%, sugar, potato starch, wheat flour, rapeseed oil, salt, acidity regulator: citric acid, spices; paprika 2.5%, onion.  **Nutrition Facts** per 100g: Energy value 617 kj/148 kcal Fat: 9.5 g, including saturated fat 2.1 g; Carbohydrates: 4.2 g, including sugars 3.6 g; Fibre: 0.9 g, Protein: 11 g, Salt: 0.95 g; Omega-3 fatty acids: 2.2 g, including EPA: 700 mg, DHA: 800 mg. | C Yellow | 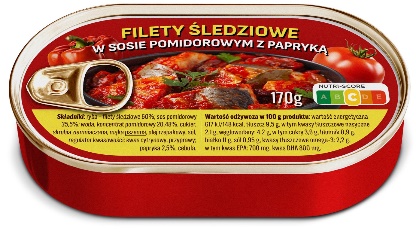 |
| 12 |  | Herring fillets in vegetable oil | **Ingredients**: Fish - herring fillets 70%, rapeseed oil 29%, salt.  **Nutrition Facts** per 100 g: Energy value 1496 kj/362 kcal Fat: 34 g, of which saturated fat 3,9 g; Carbohydrates 0 g, of which sugars 0 g; Fibre: 0 g; Protein: 14 g; Salt: 0,75 g; Omega-3 fatty acids: 3,3 g, of which EPA 200 mg, DHA 500 mg | C Yellow | 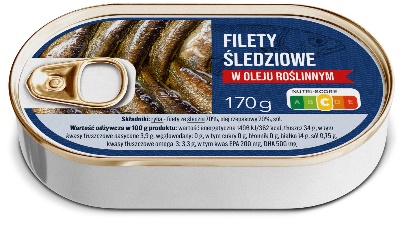 |
| 13 | 7 | Hot smoked Atlantic salmon - bellies with peel | **Ingredients**: Atlantic salmon (salmo salar) 98%, salt  **Nutrition Facts** per 100g: Energy value 1989 kj/482 kcal, fat 46 g, of which saturated fat 15 g; carbohydrates <0.5 g, of which sugars <0.5 g; protein 16 g, salt 1.8 g; Omega-3 fatty acids: 7.8 g, of which DHA: 2.2 g, EPA: 1.5 g | E  Dark Orange | 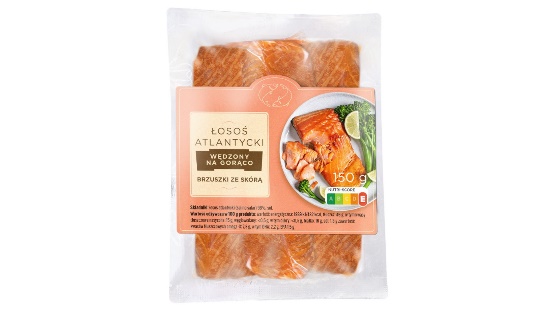 |
| 14 |  | Fillets of sardines in extra virgin olive oil | **Ingredients**: Sardine fillets (sardina pilchardus), extra virgin olive oil 19%, salt  **Nutrition Facts** per 100g: Energy value 855 kj/205 kcal, fat 13 g, of which saturated fat 3.3 g, protein 22 g, salt 1.0 g. | B  Green | 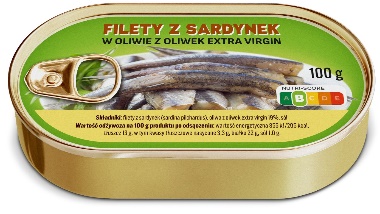 |
| 15 | 8 | Serrano pork ham, dry-cured, matured | **Ingredients**: Pork ham, salt, sugar, preservatives: potassium nitrate, sodium nitrite, dextrose, antioxidant: sodium ascorbate  **Nutrition Facts** per 100g: Energy value 903 kj/216 kcal, fat 10 g, of which saturated fat 3.9 g; carbohydrates 1 g, of which sugars 1 g; protein 30 g, salt 5.9 g | E  Dark Orange | 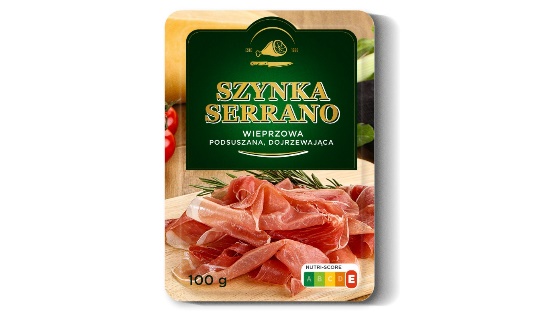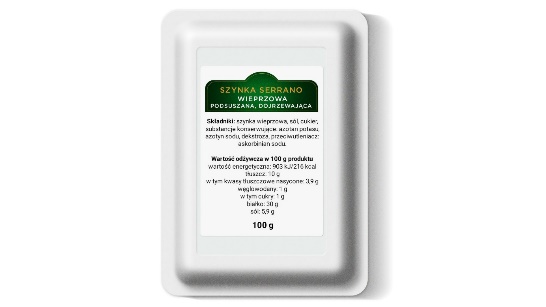 |
| 16 |  | Pork ham | **Ingredients**: Pork ham meat 82%, water, salt, stabilizers: triphosphates, carrageenan, soy protein, maltodextrin, glucose, sugar, sunflower protein hydrolysate, antioxidant: sodium ascorbate, yeast extract, flavors, spice extracts, preservative: sodium nitrite  **Nutrition Facts** per 100g: Energy value 394 kj/93 kcal, fat 2 g, of which NKT 0.7 g; carbohydrates 1.5 g, of which sugars 0.5 g; protein 17.2 g, fibre 0.2 g, salt 1.98 g | C Yellow | 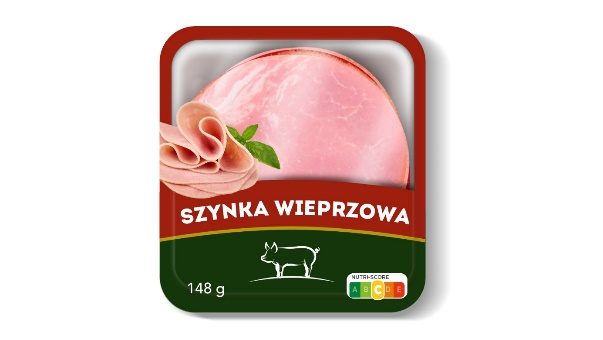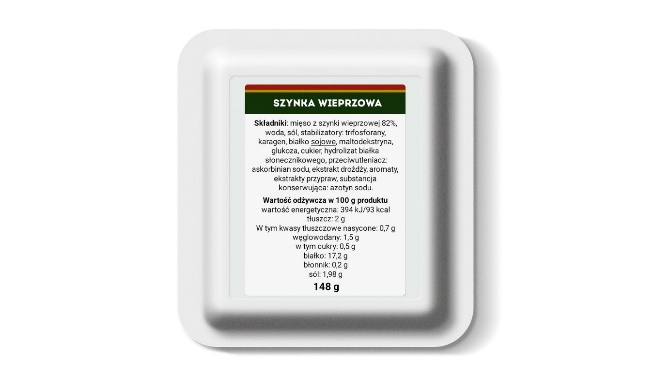 |
