# Supplementary material for: Assessment of the influence of Nutri-Score on Polish consumer Choices - Insights from the nationwide, Cross-sectional study
Source: Sci Rep. 2025 Aug 11;15:29422. doi: 10.1038/s41598-025-14033-9 (PMC12339969; doi:10.1038/s41598-025-14033-9)
Supplement: Supplementary file 2 — Supplementary Material 2 [file 41598_2025_14033_MOESM2_ESM.docx]

| 1 | Creamy yoghurt with strawberries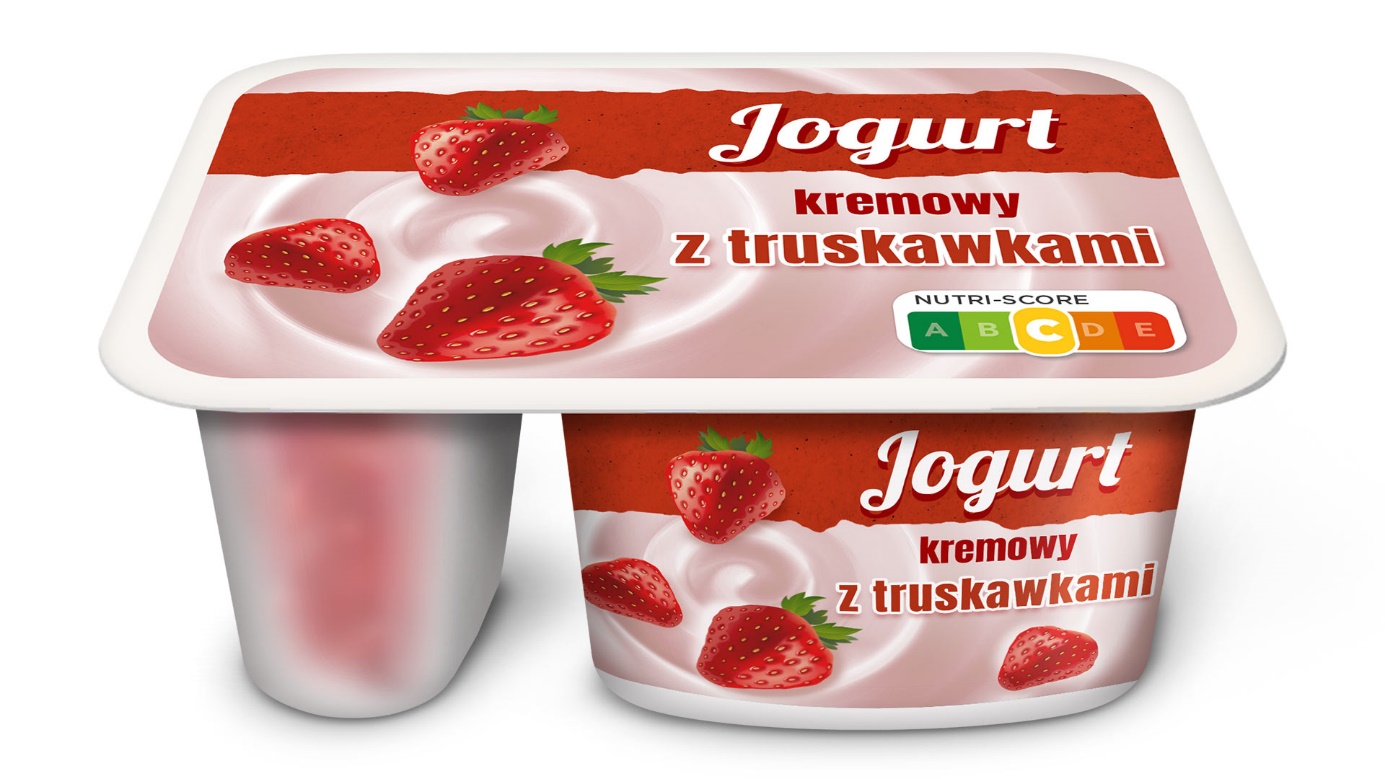 | C – Yellow |
| --- | --- | --- |
| 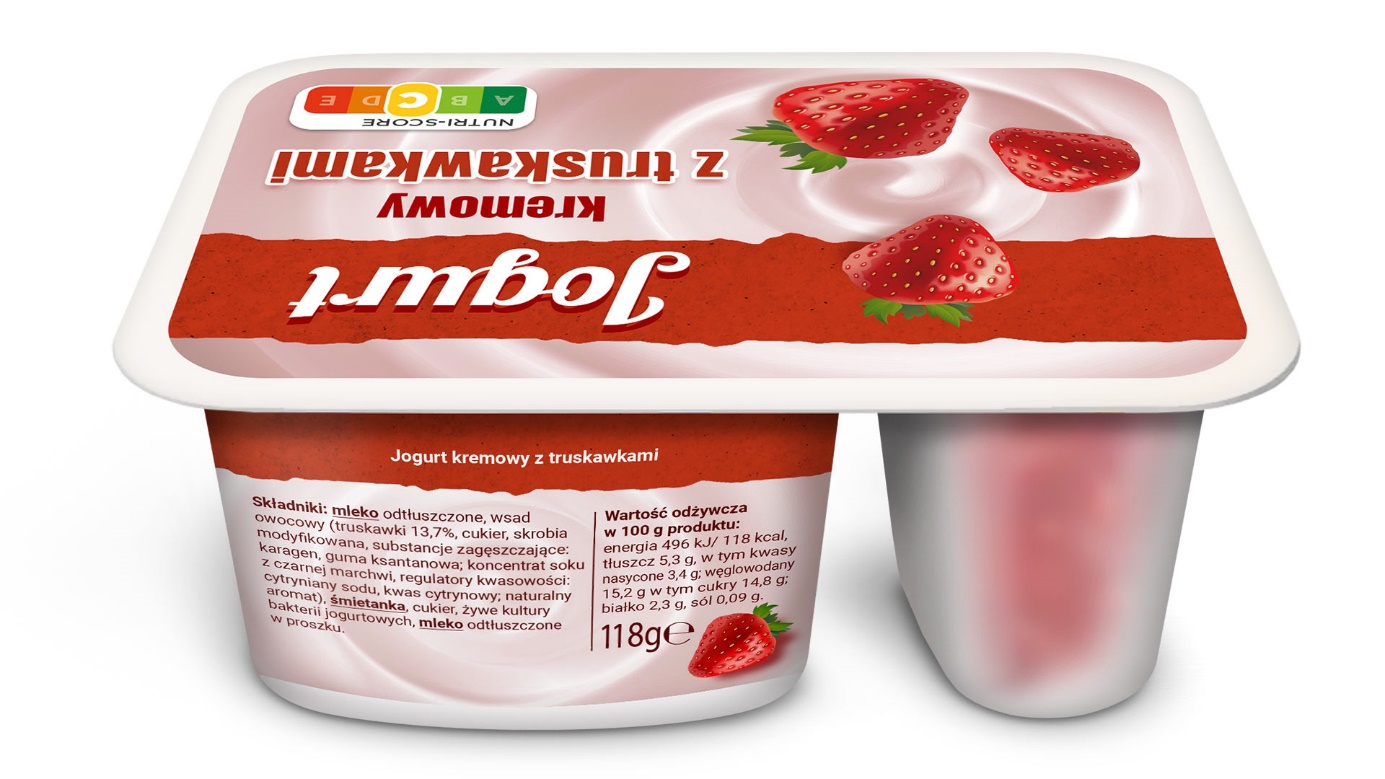 | | |
| 2 | Creamy yoghurt with chocolate-covered flakes | C – Yellow |
| 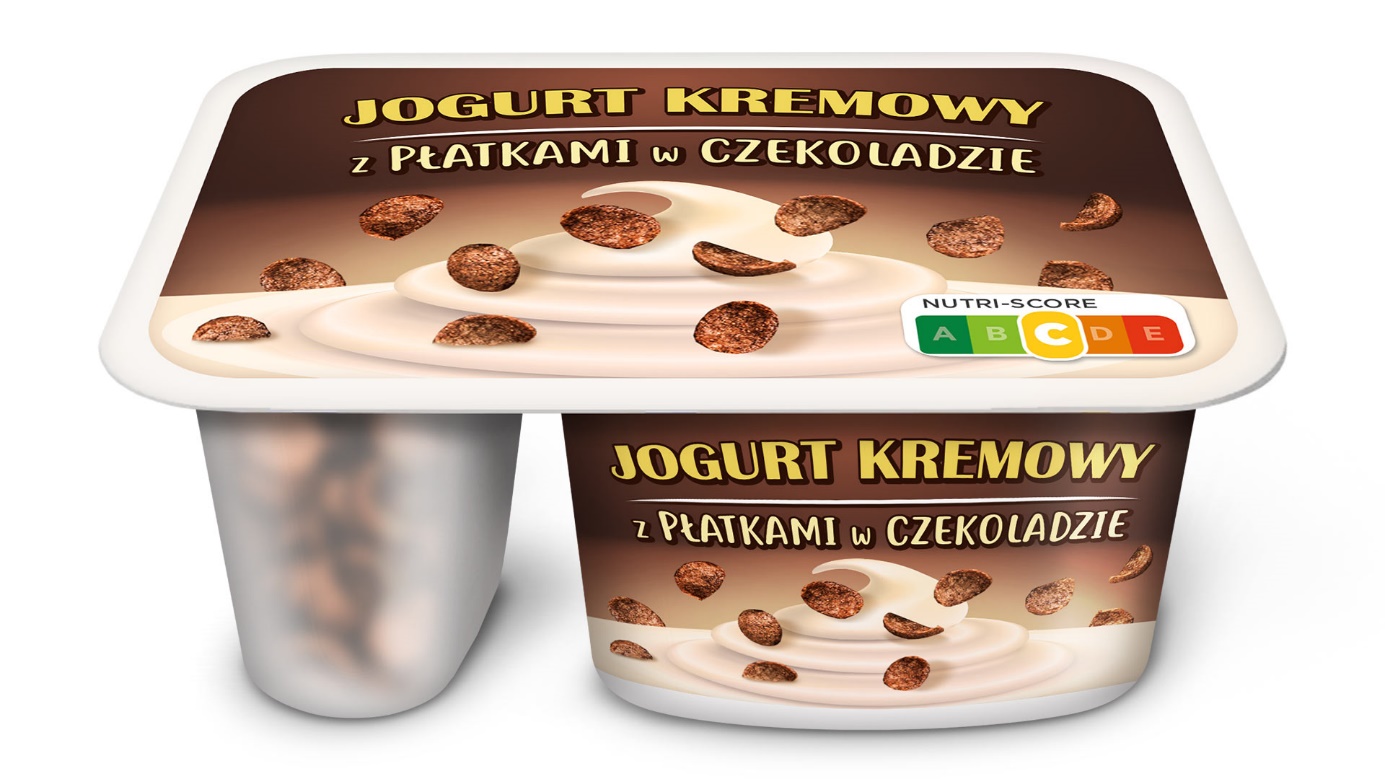  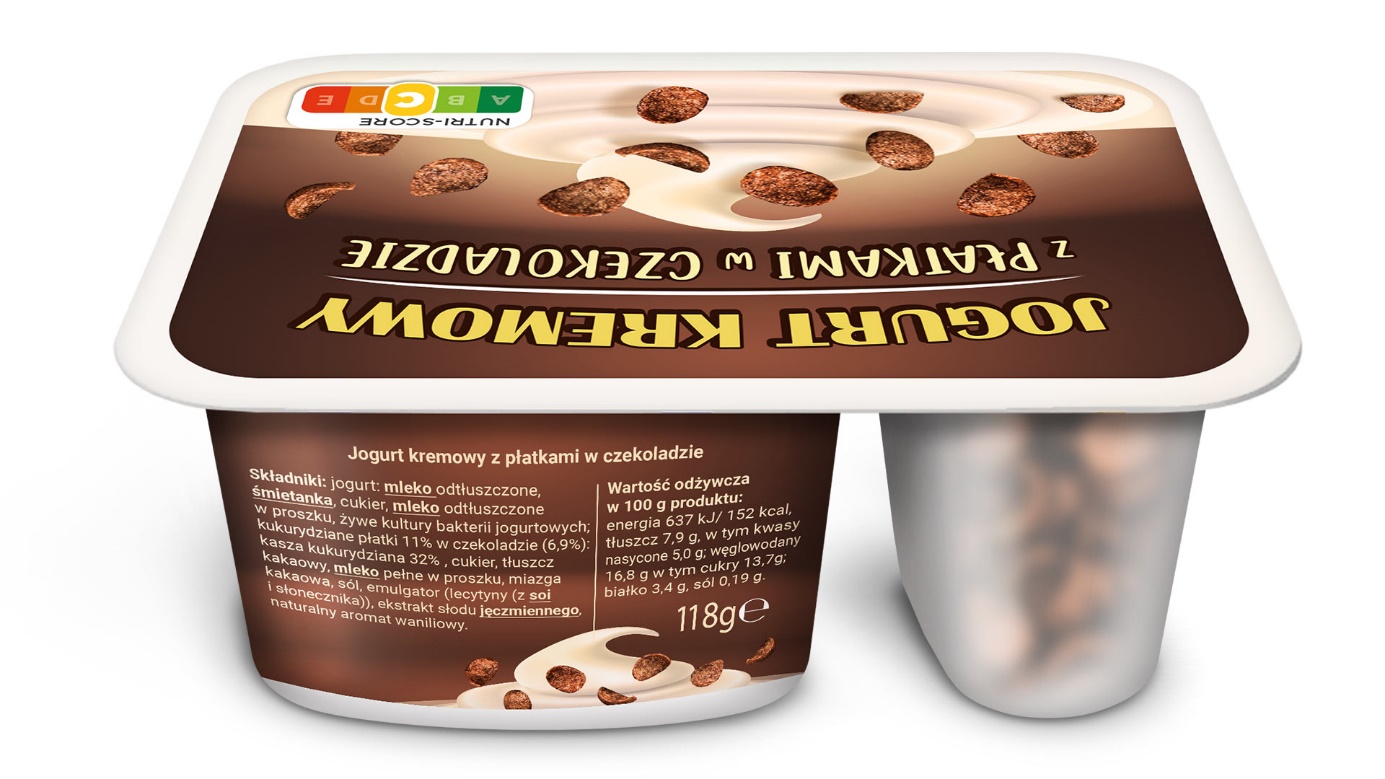 | | |
| 3 | Multi-grain bread. Whole-grain rye bread, sliced | A – Dark Green |
| 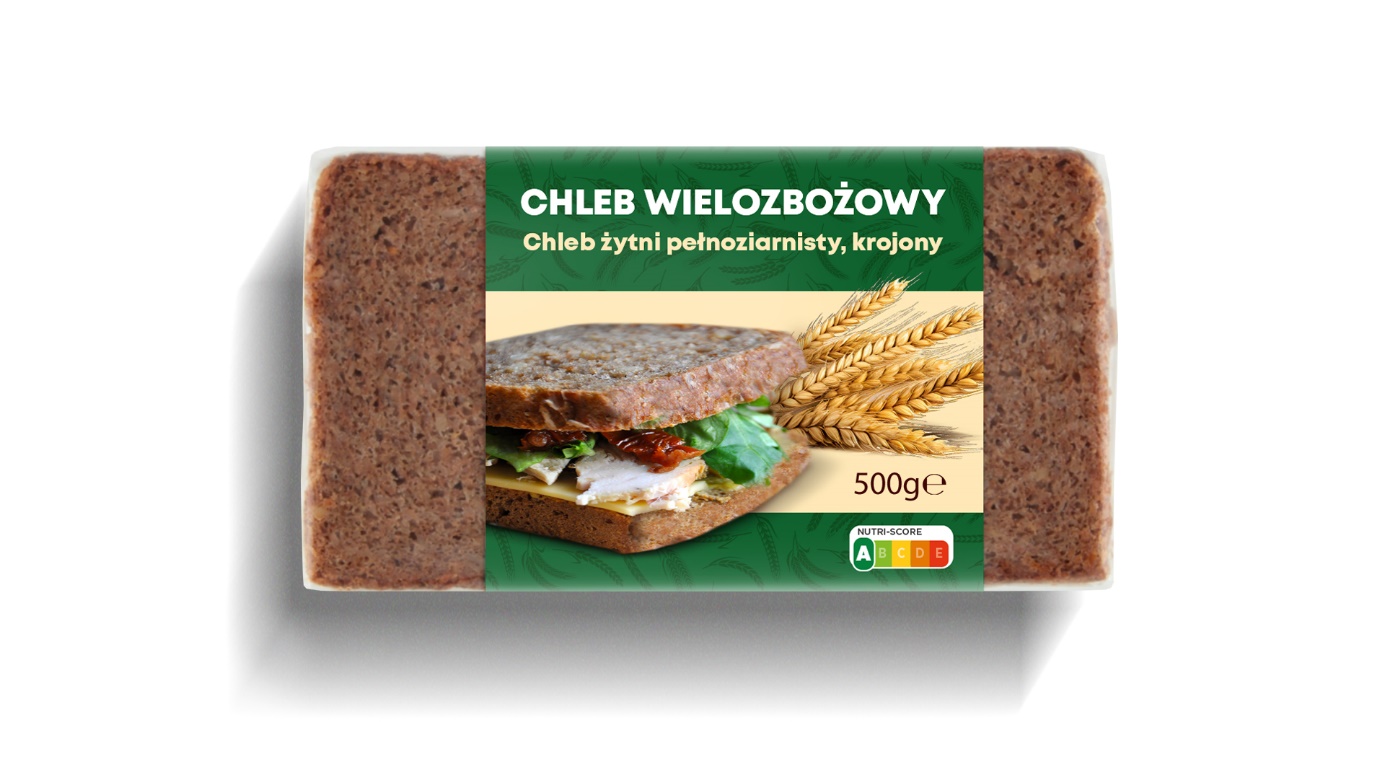  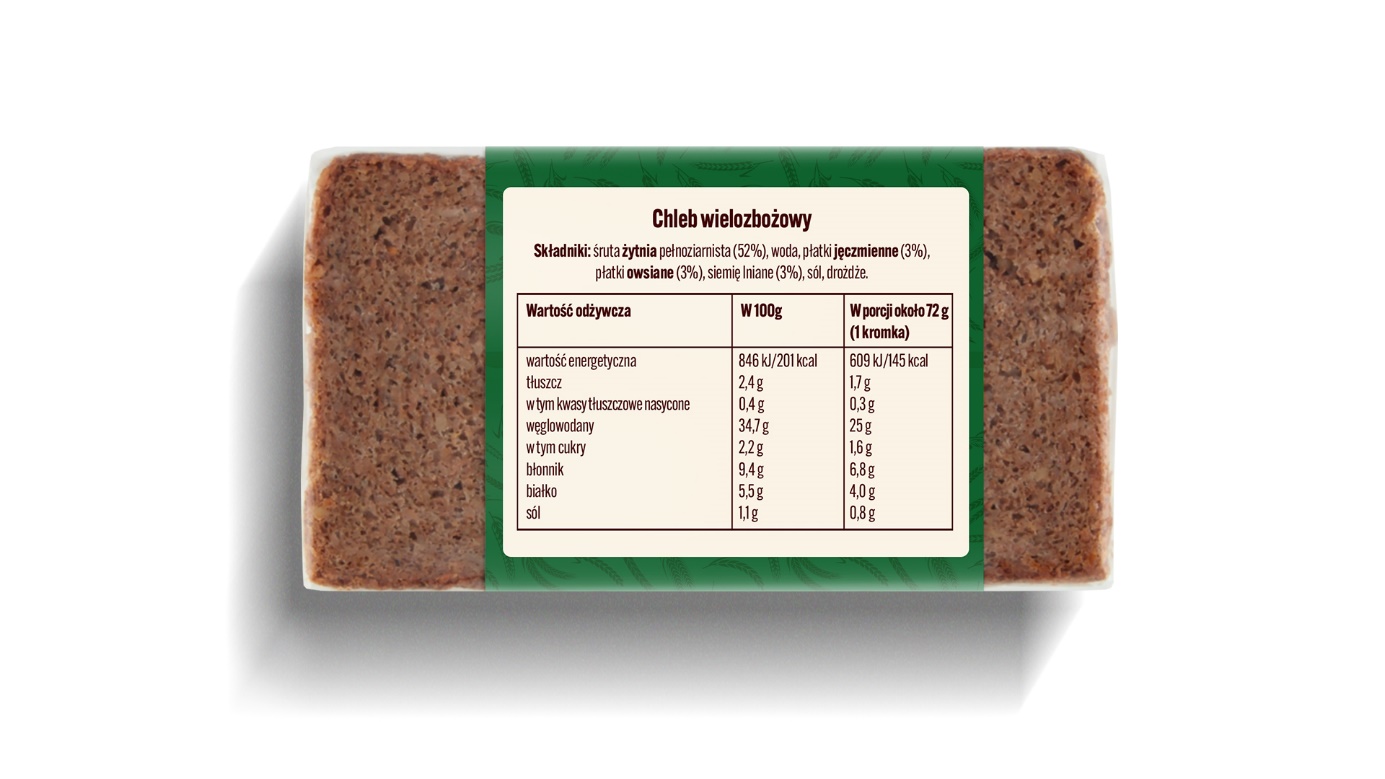 | | |
| 4 | Whole-grain toast bread | B – Green |
| 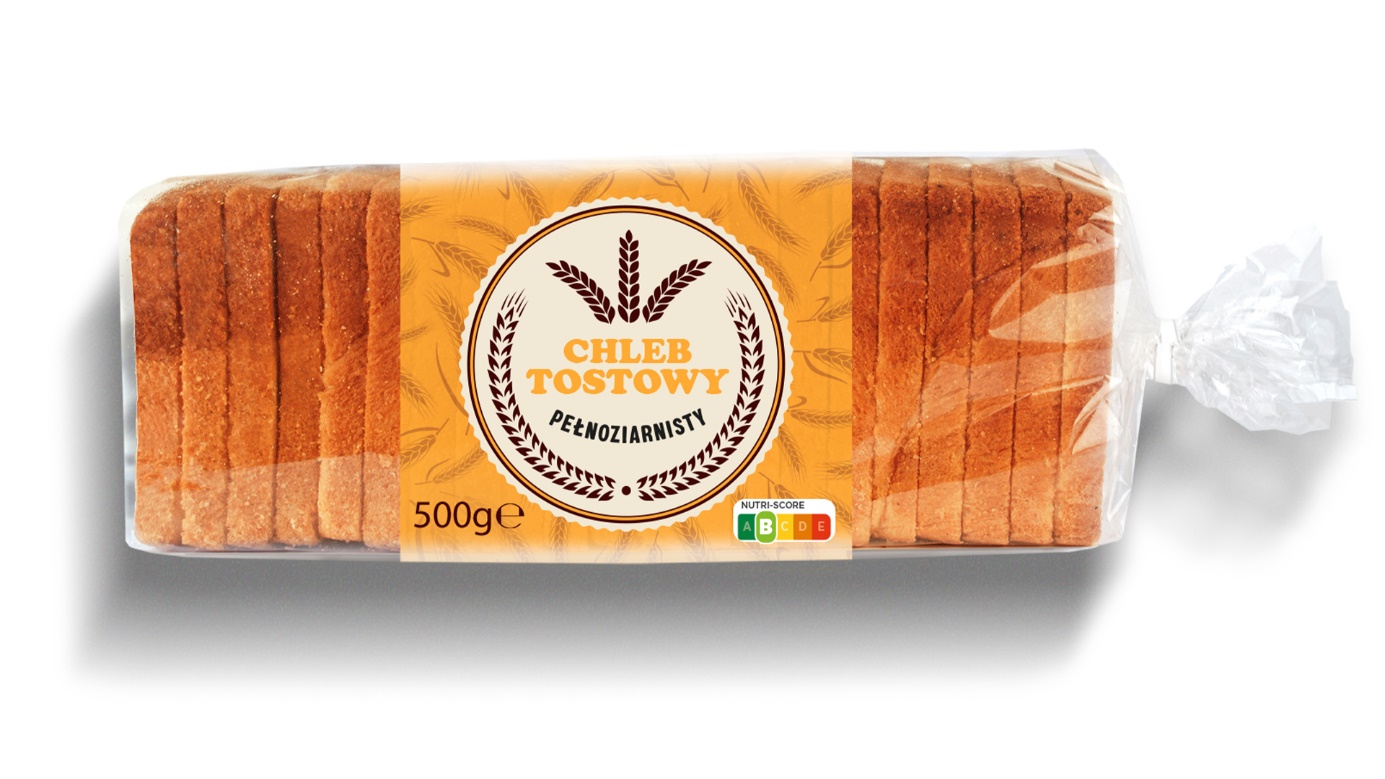  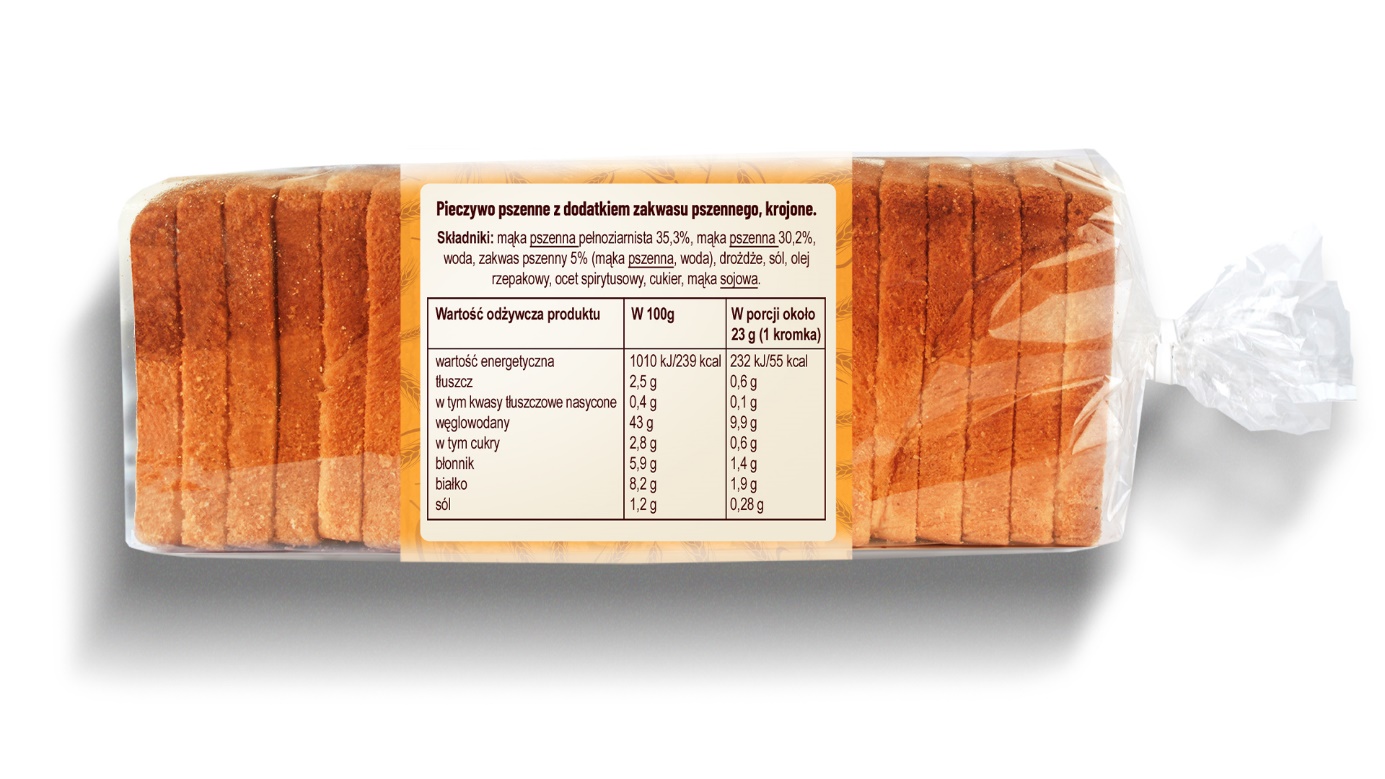 | | |
| 5 | Penne rigate wholegrain pasta | A – Dark Green |
| 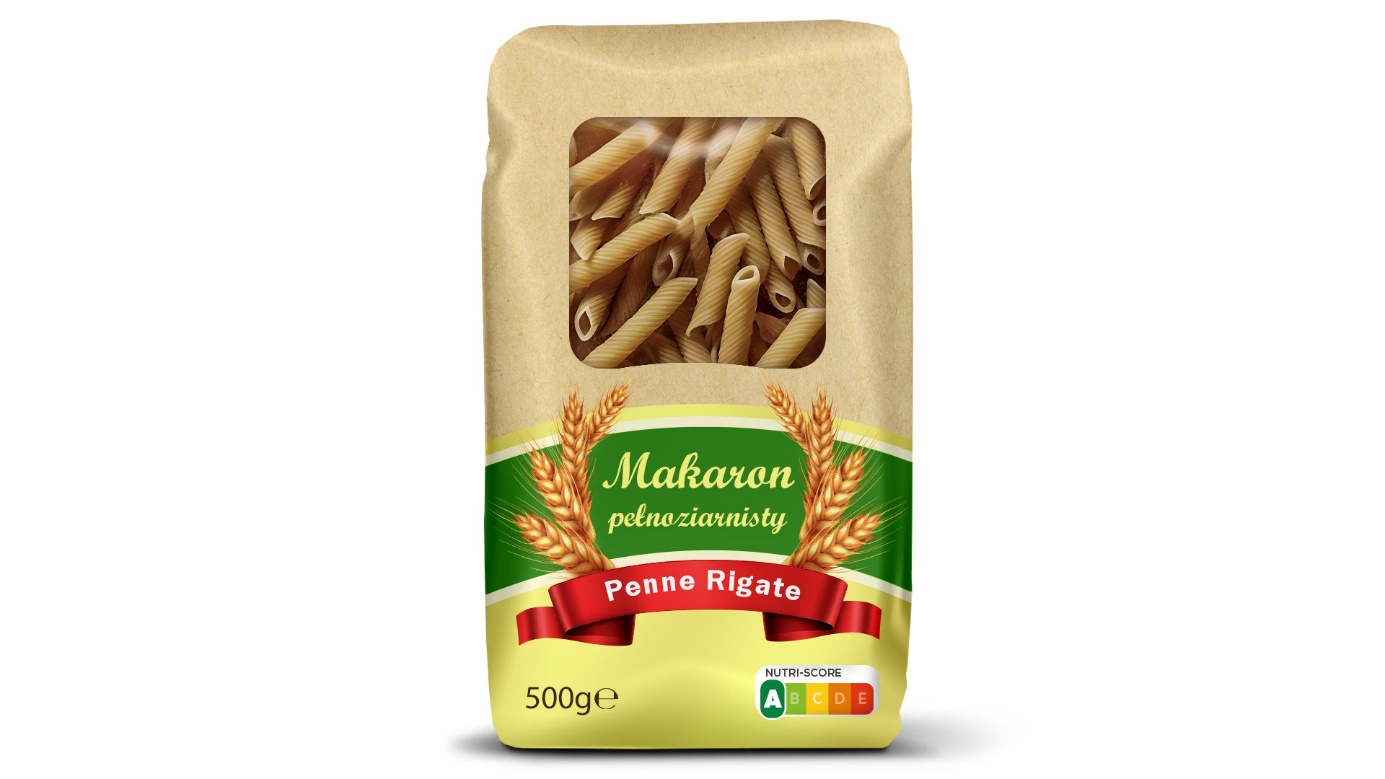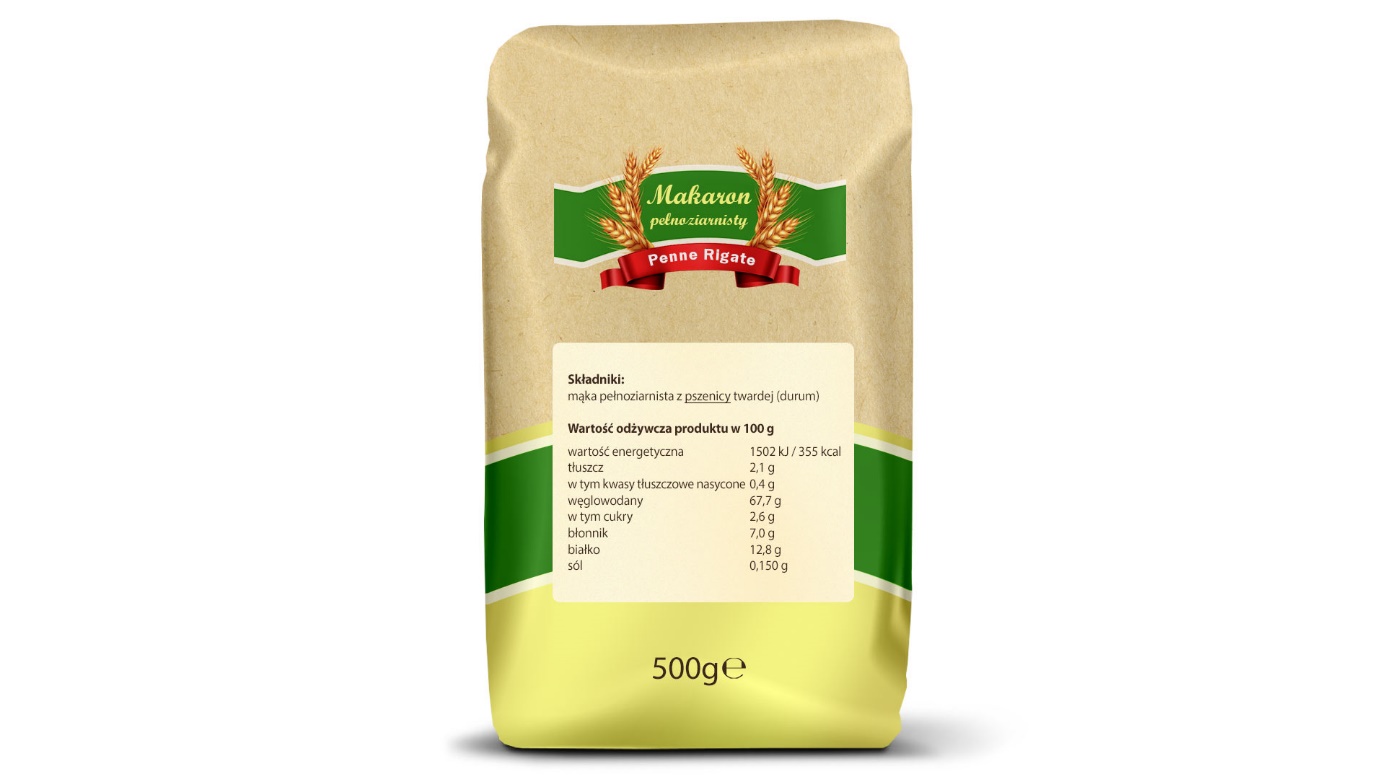 | | |
| 6 | Penne rigate pasta | A – Dark Green |
| 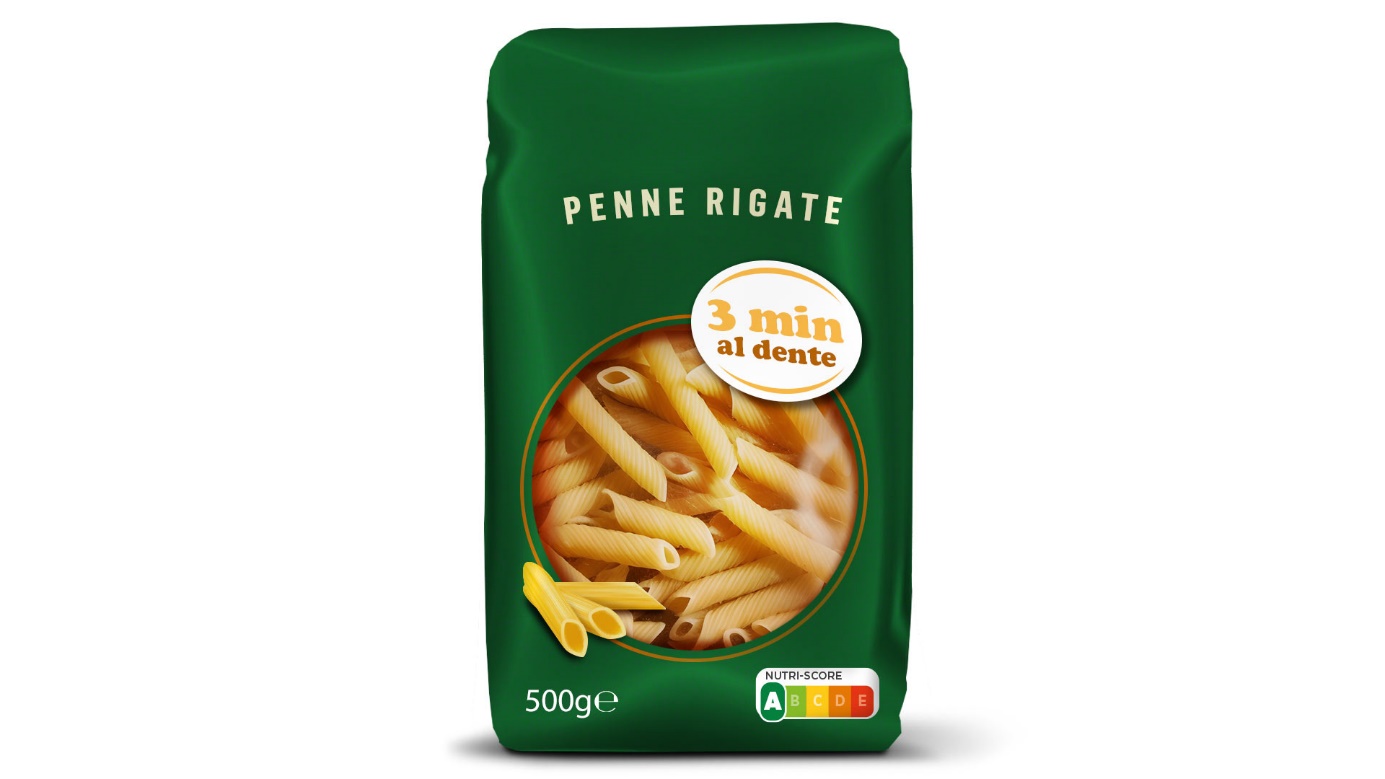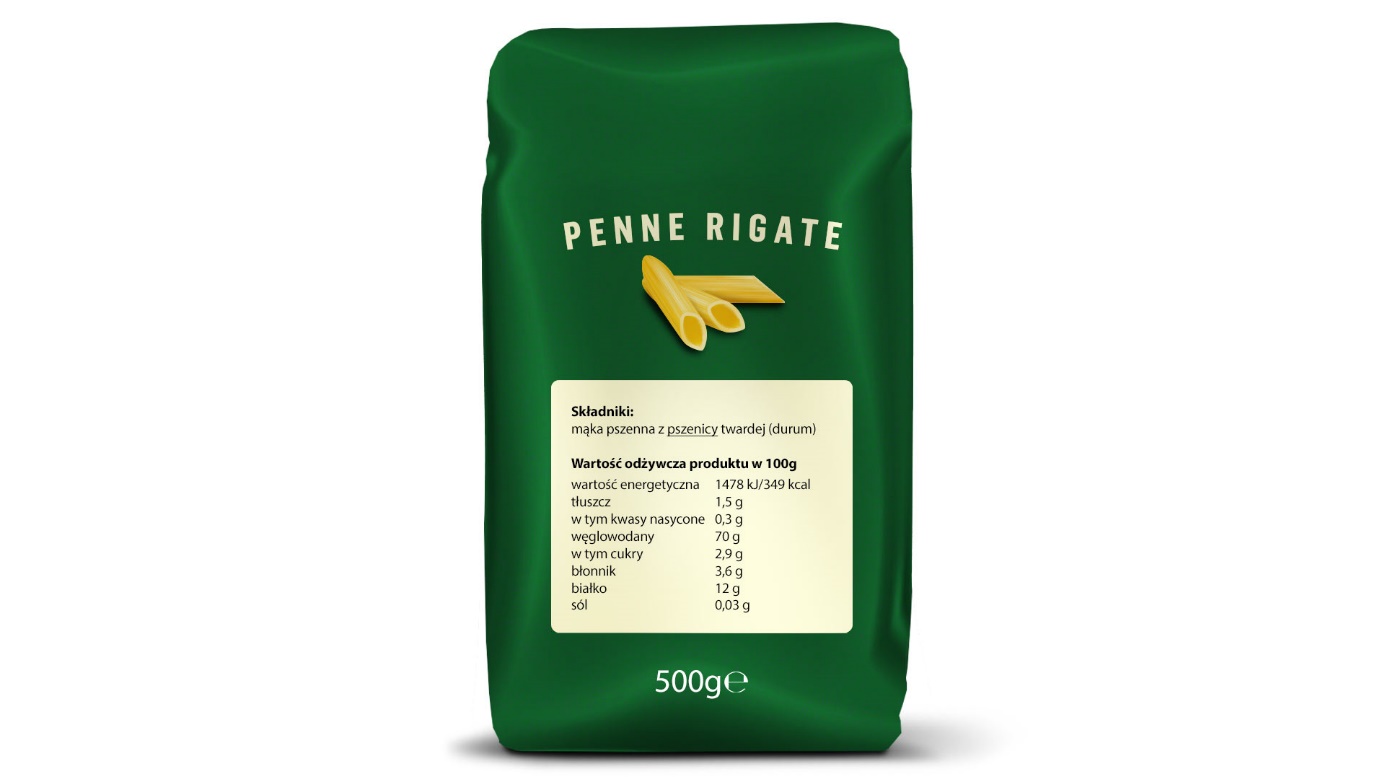 | | |
| 7 | Apple carrot raspberry Juice | C – Yellow |
| 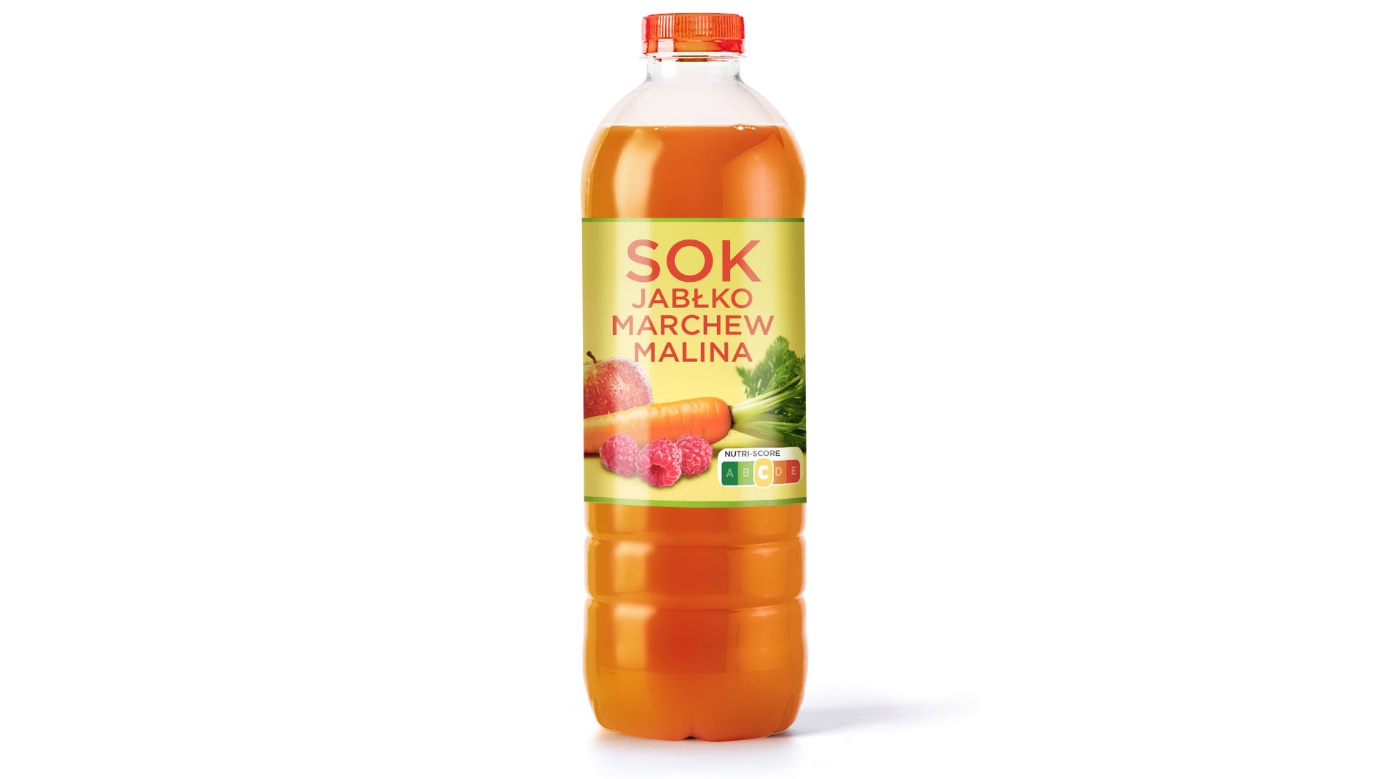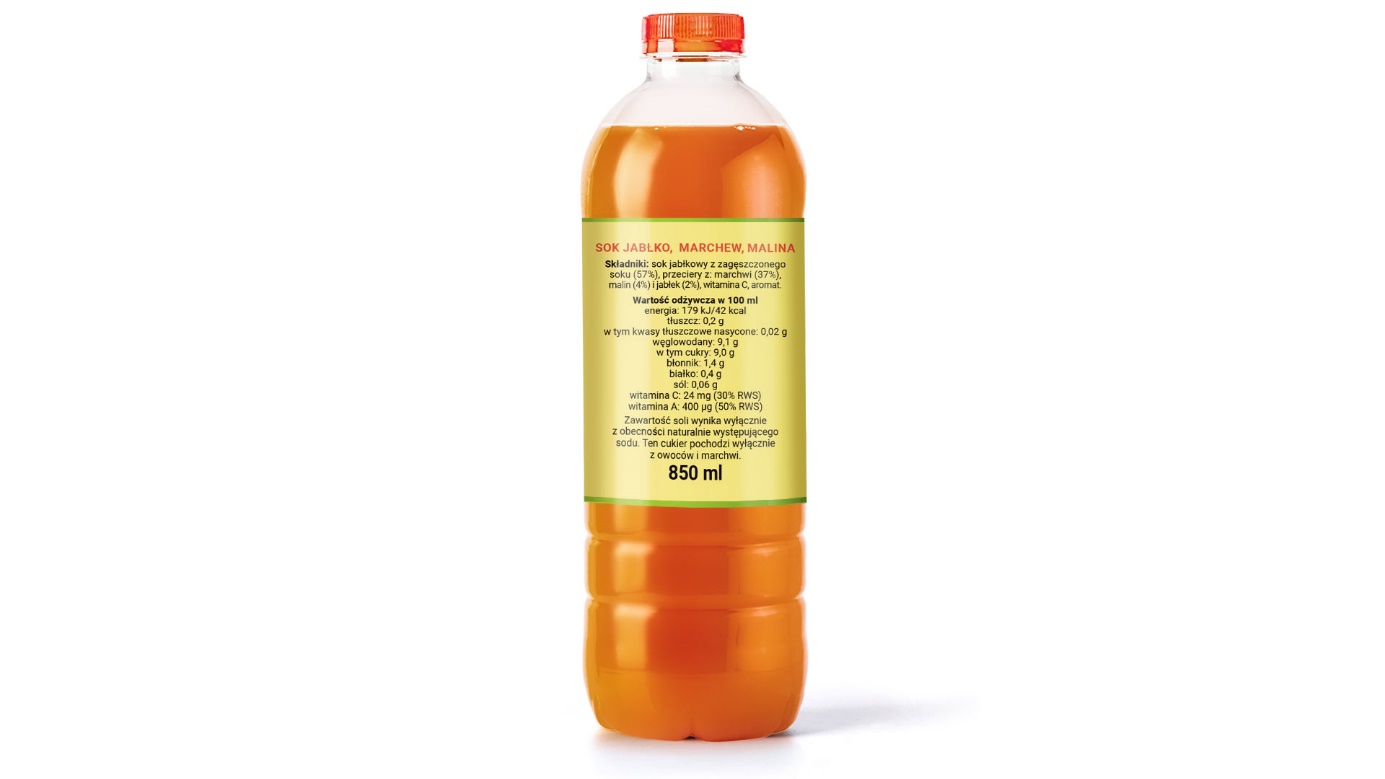 | | |
| 8 | Tea drink | C – Yellow |
| 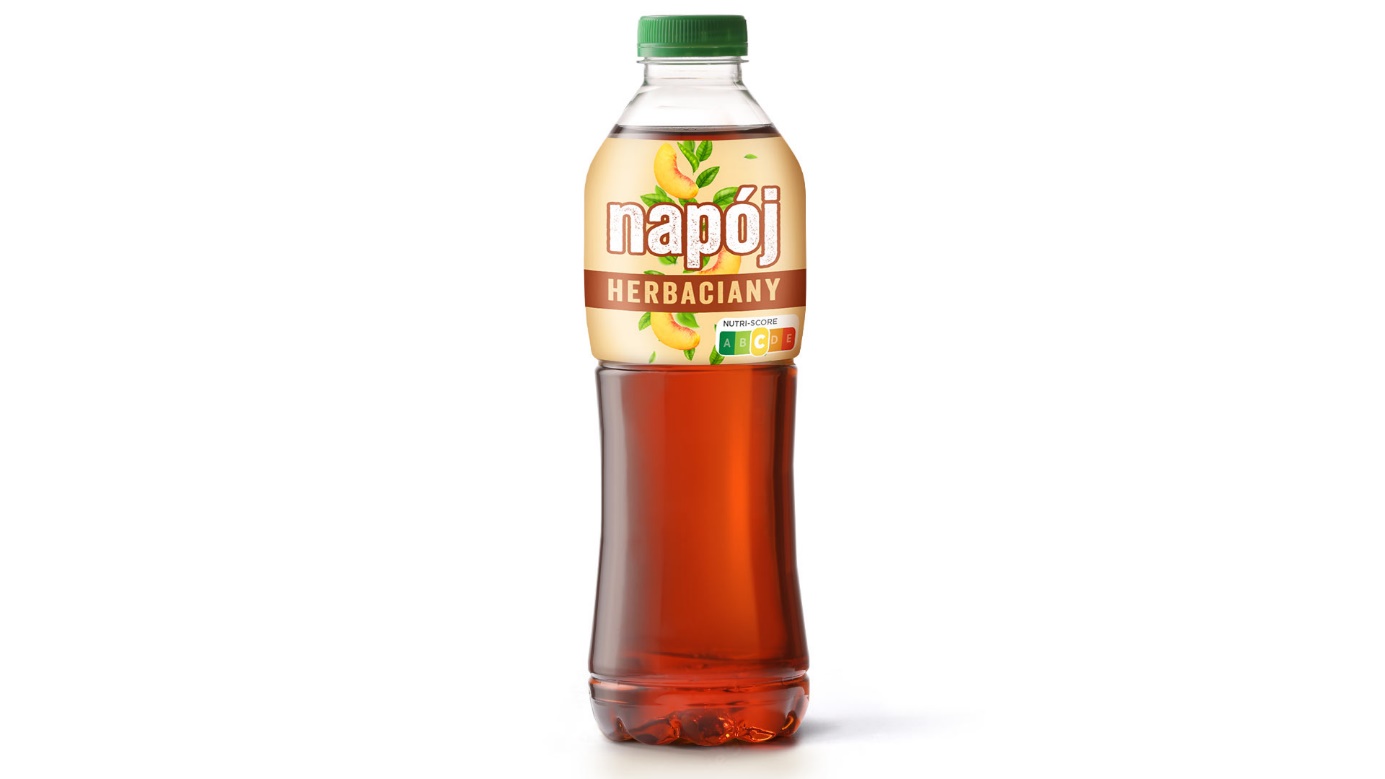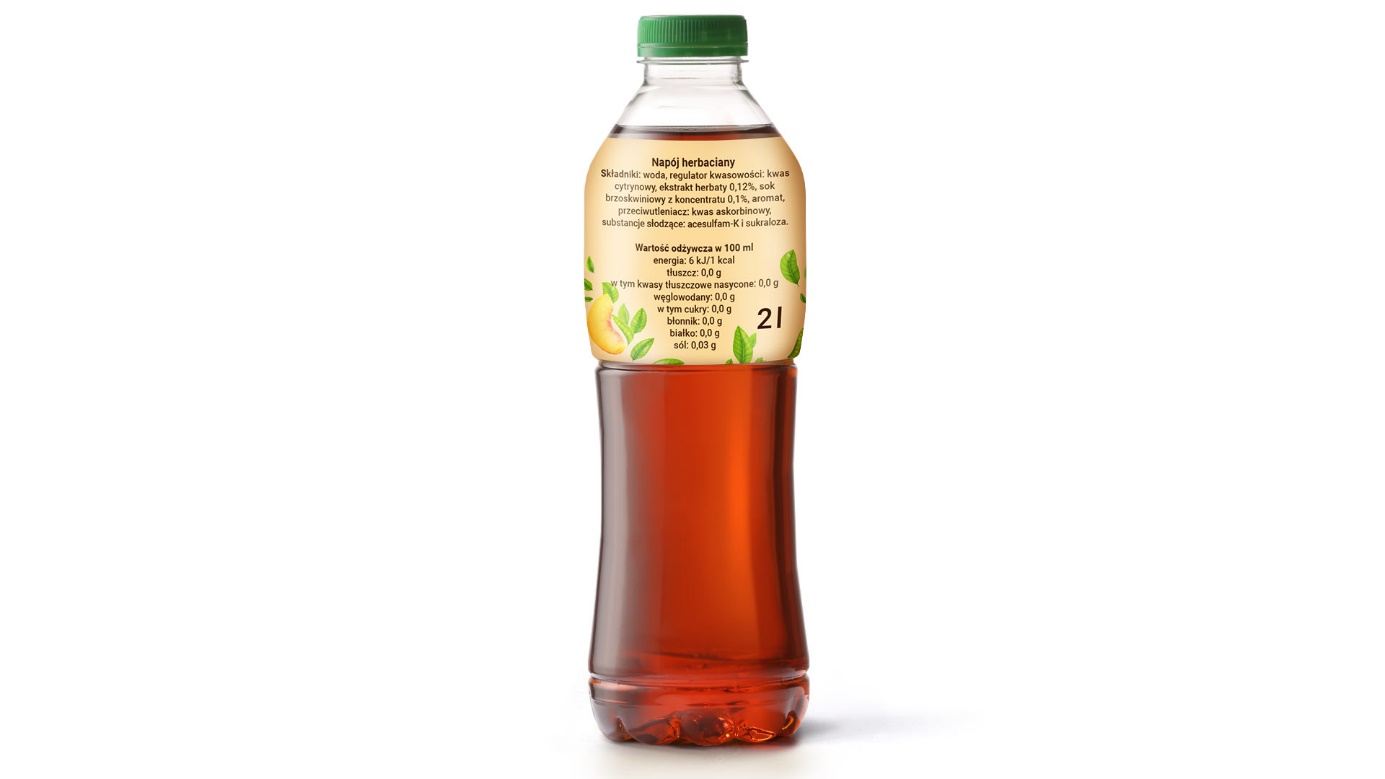 | | |
| 9 | Muesli: Fruit, flaxseed, pumpkin seeds | C – Yellow |
| 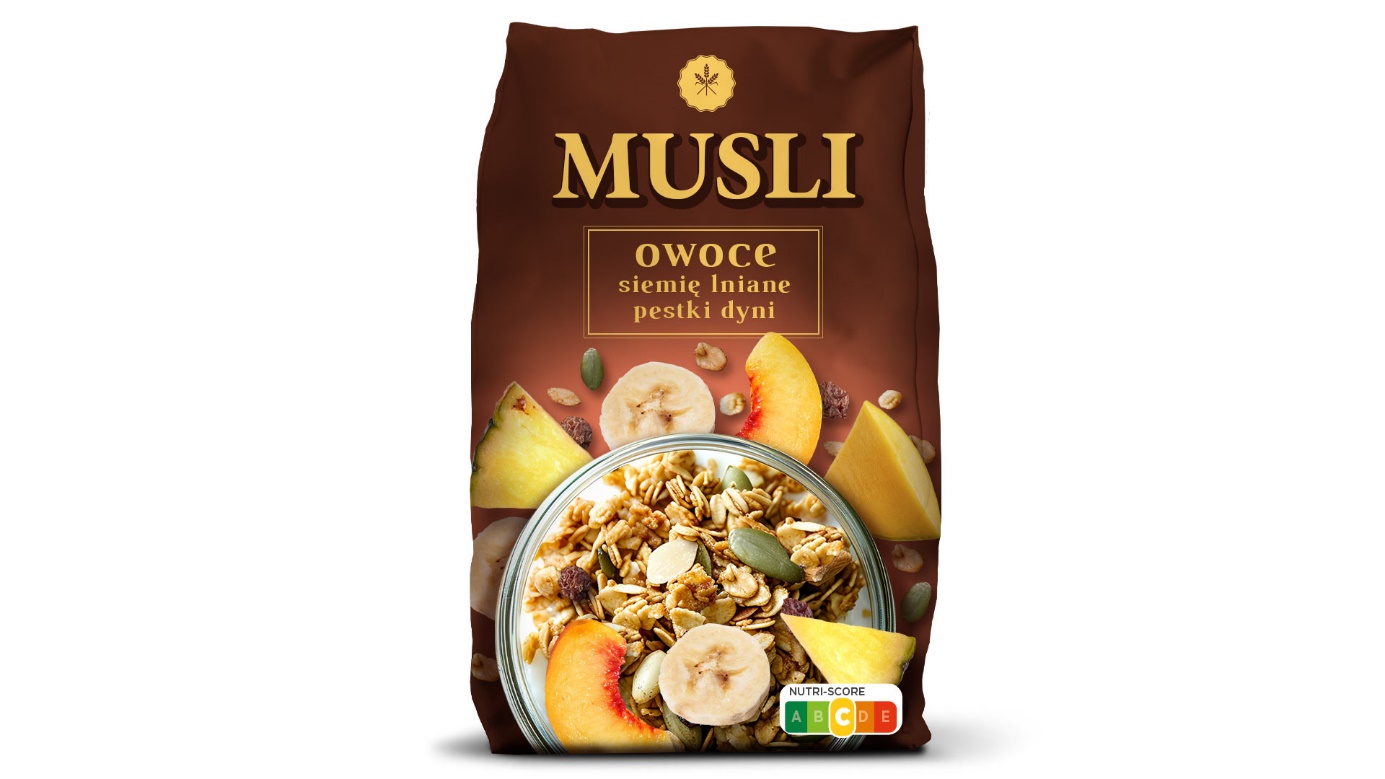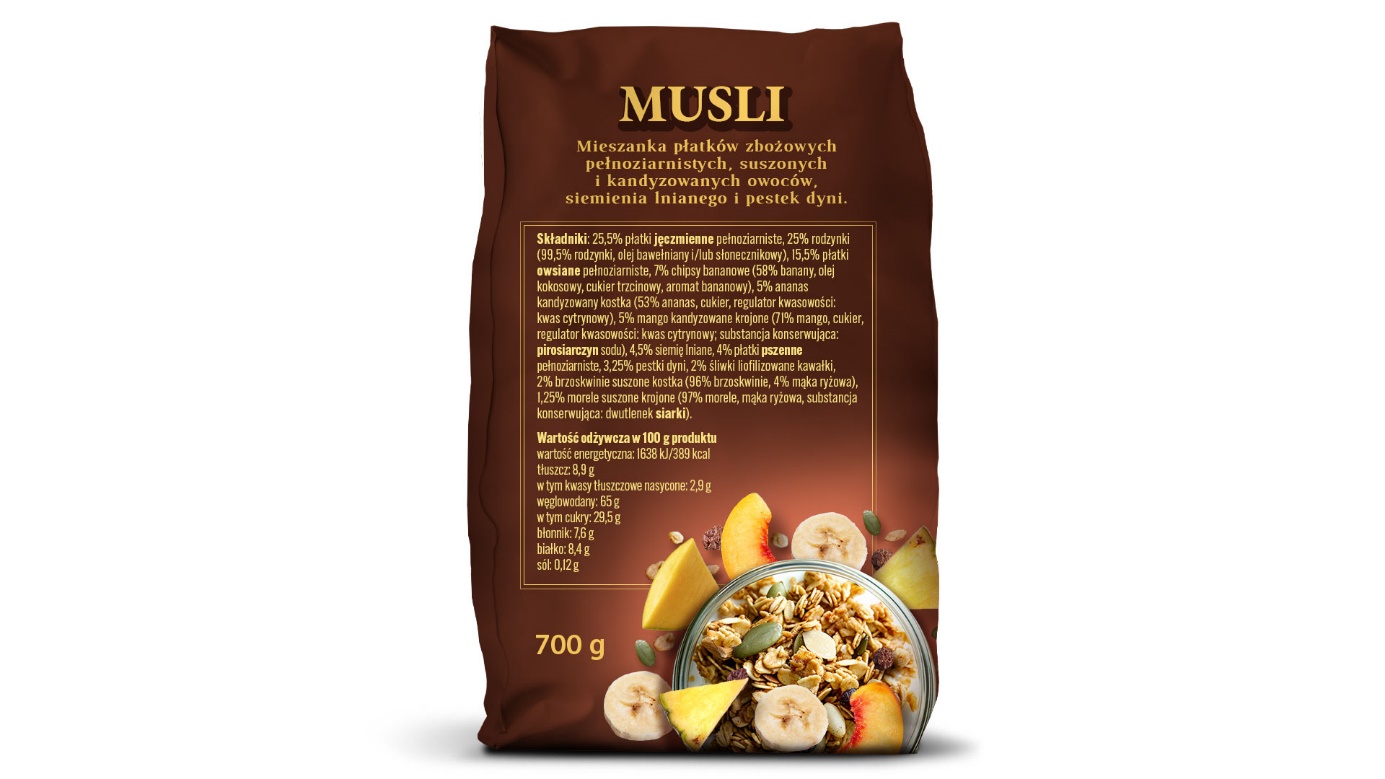 | | |
| 10 | Chocolate-flavoured cereal in the shape of balls | C – Yellow |
| 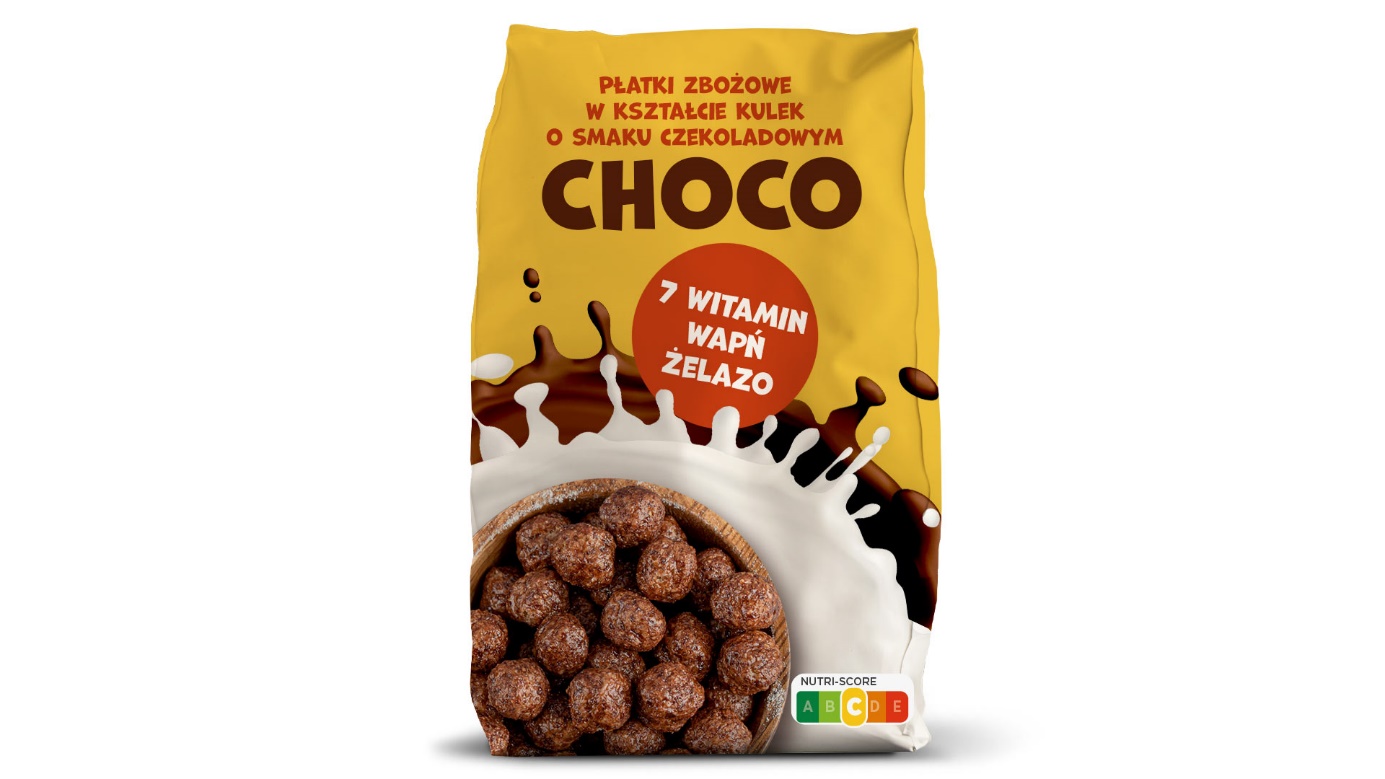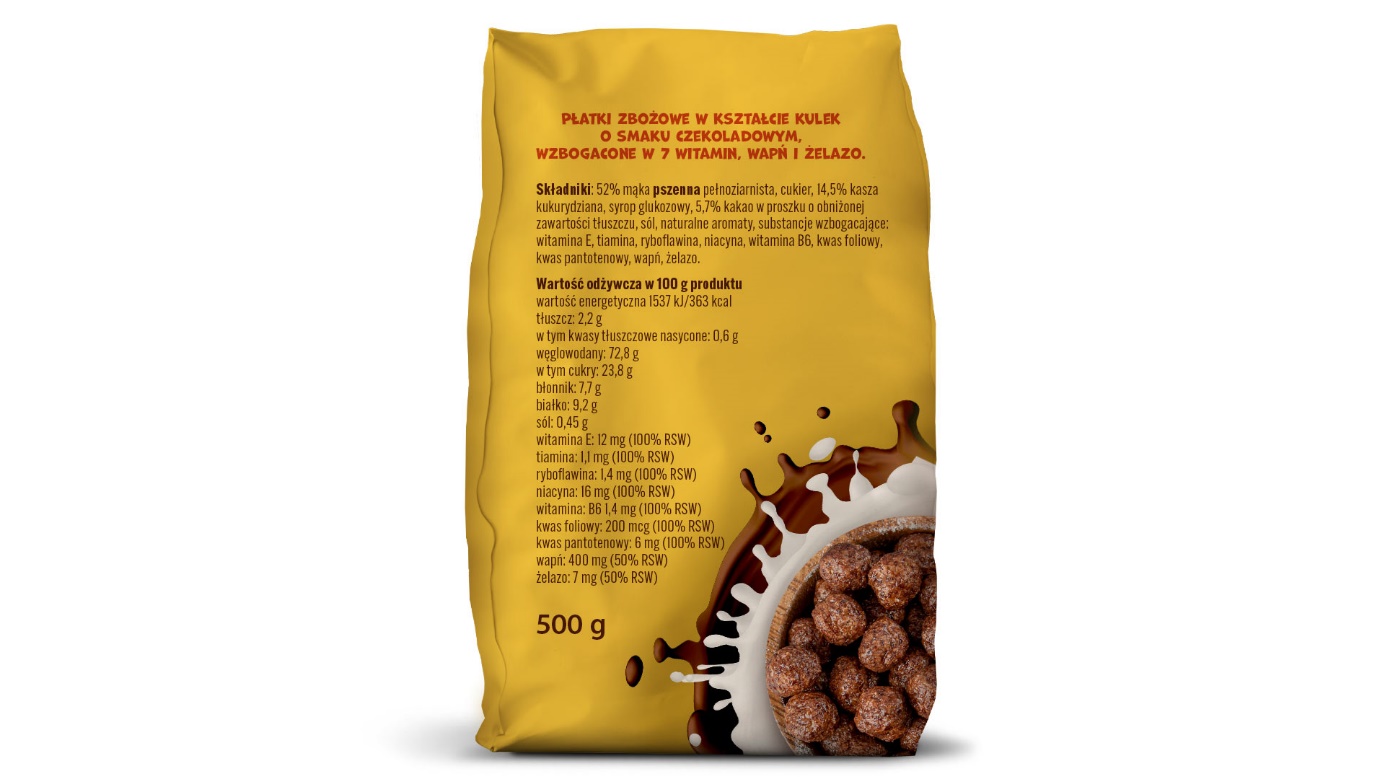 | | |
| 11 | Herring fillets in tomato sauce with paprika | C – Yellow |
| 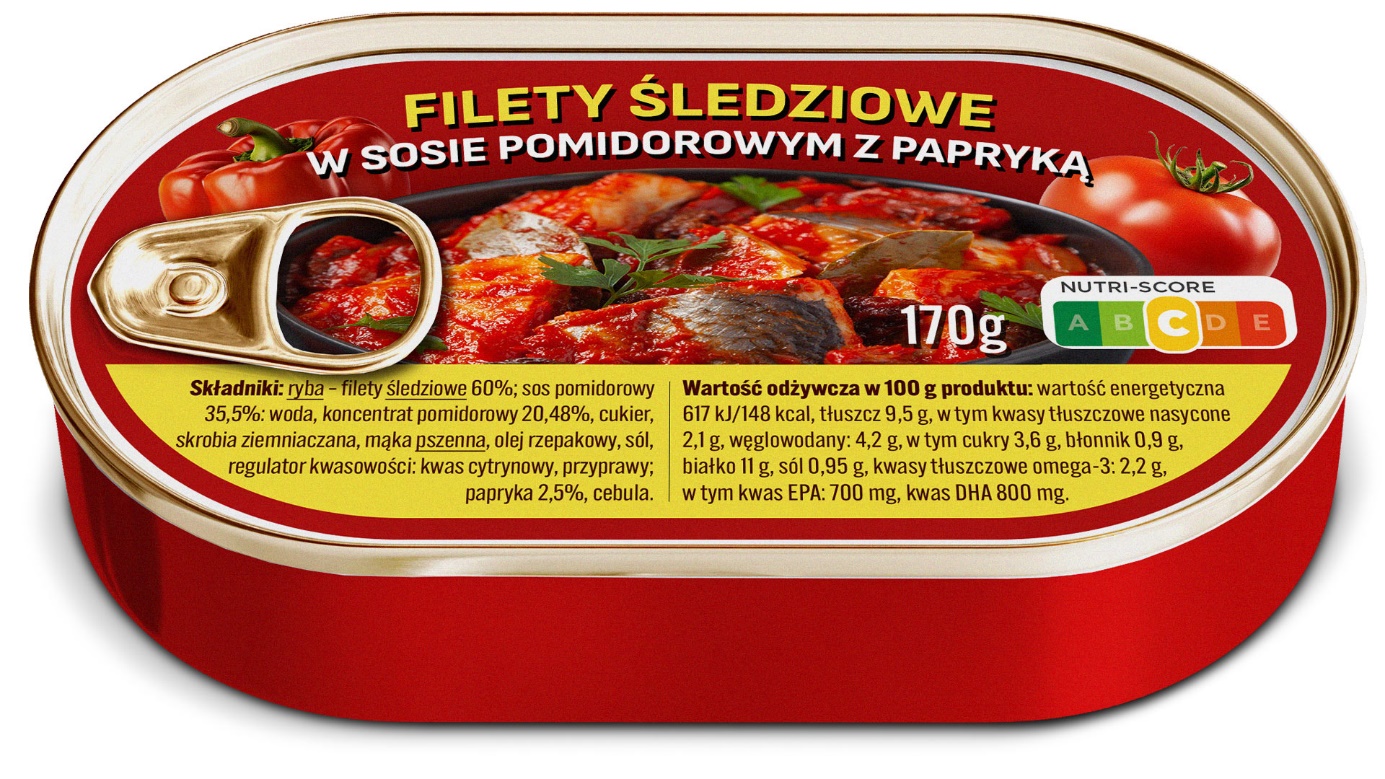 | | |
| 12 | Herring fillets in vegetable oil | C – Yellow |
| 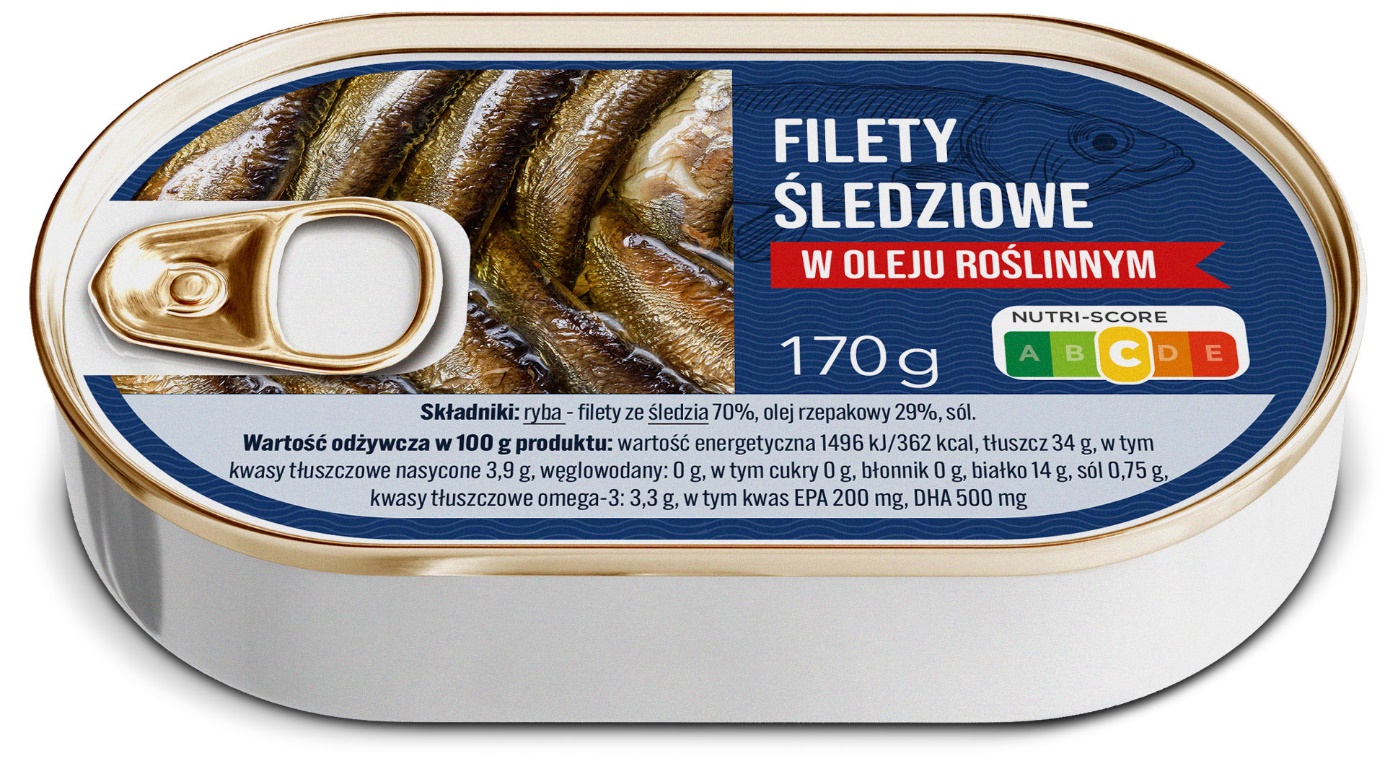 | | |
| 13 | Hot smoked Atlantic salmon - bellies with peel | E – Dark Orange |
| 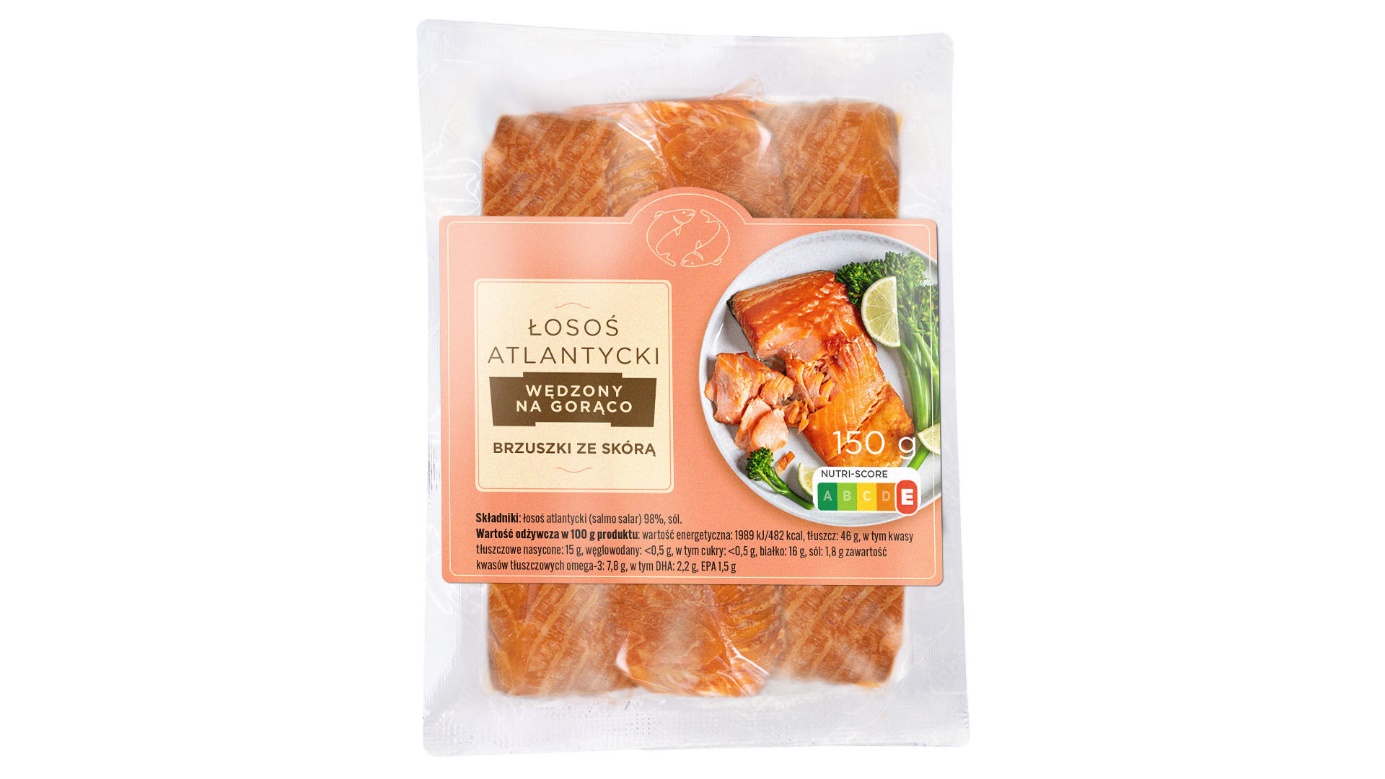 | | |
| 14 | Fillets of sardines in extra virgin olive oil | B – Green |
| 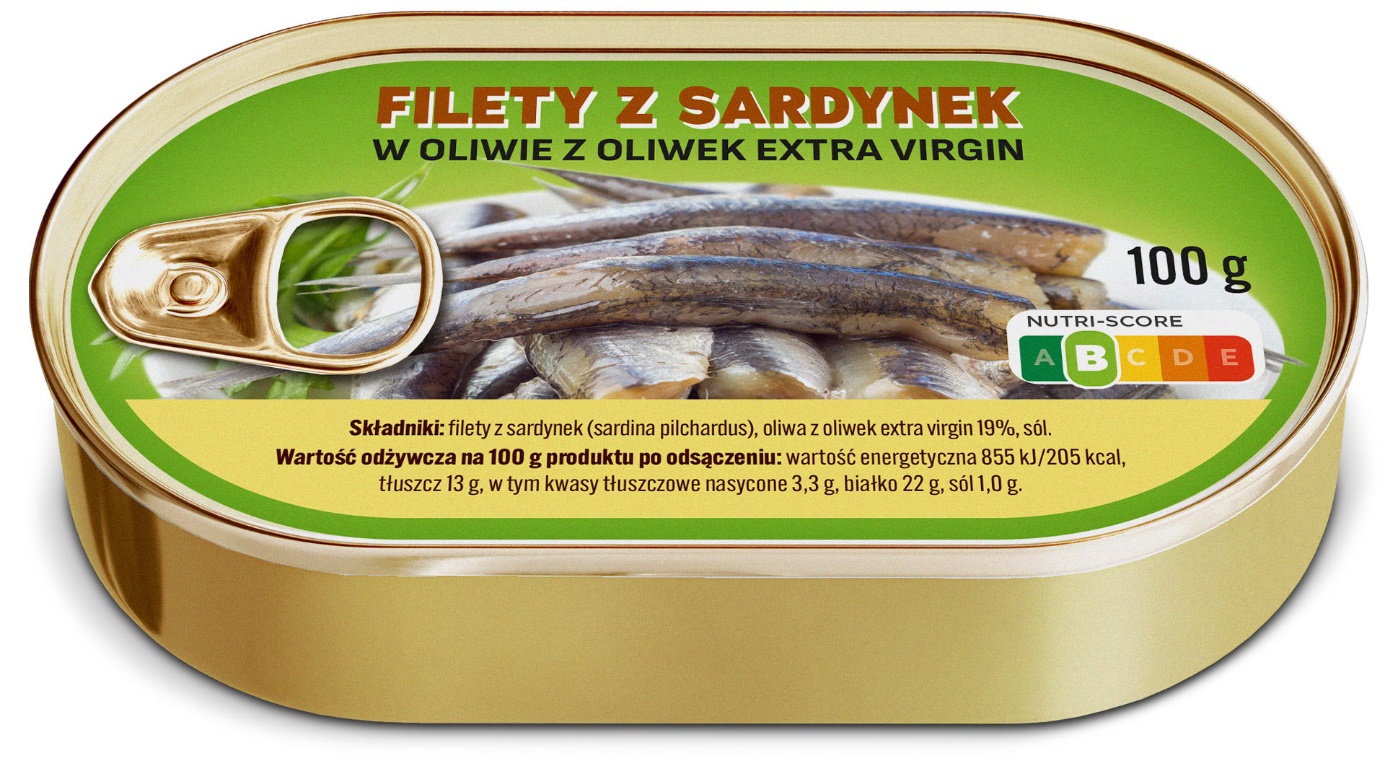 | | |
| 15 | Serrano pork ham, dry-cured, matured | E – Dark Orange |
| 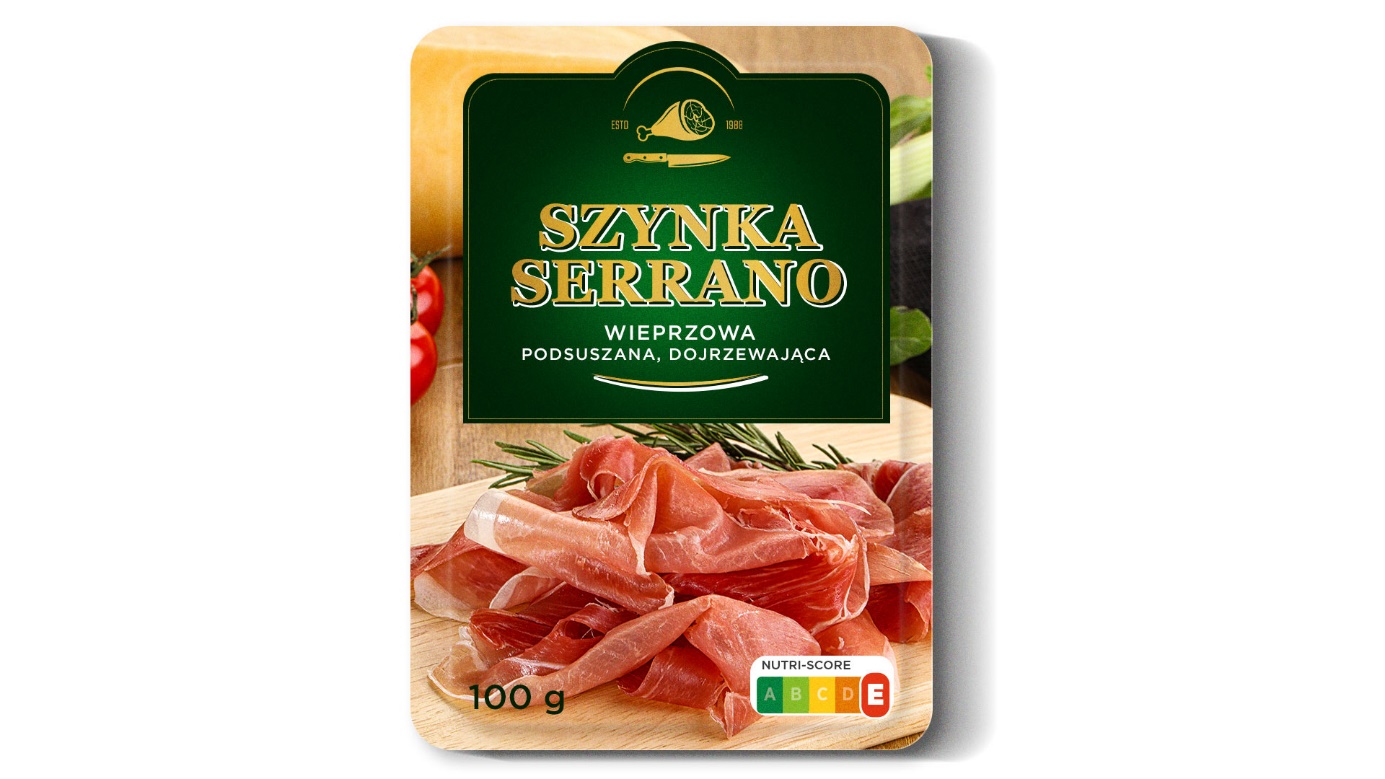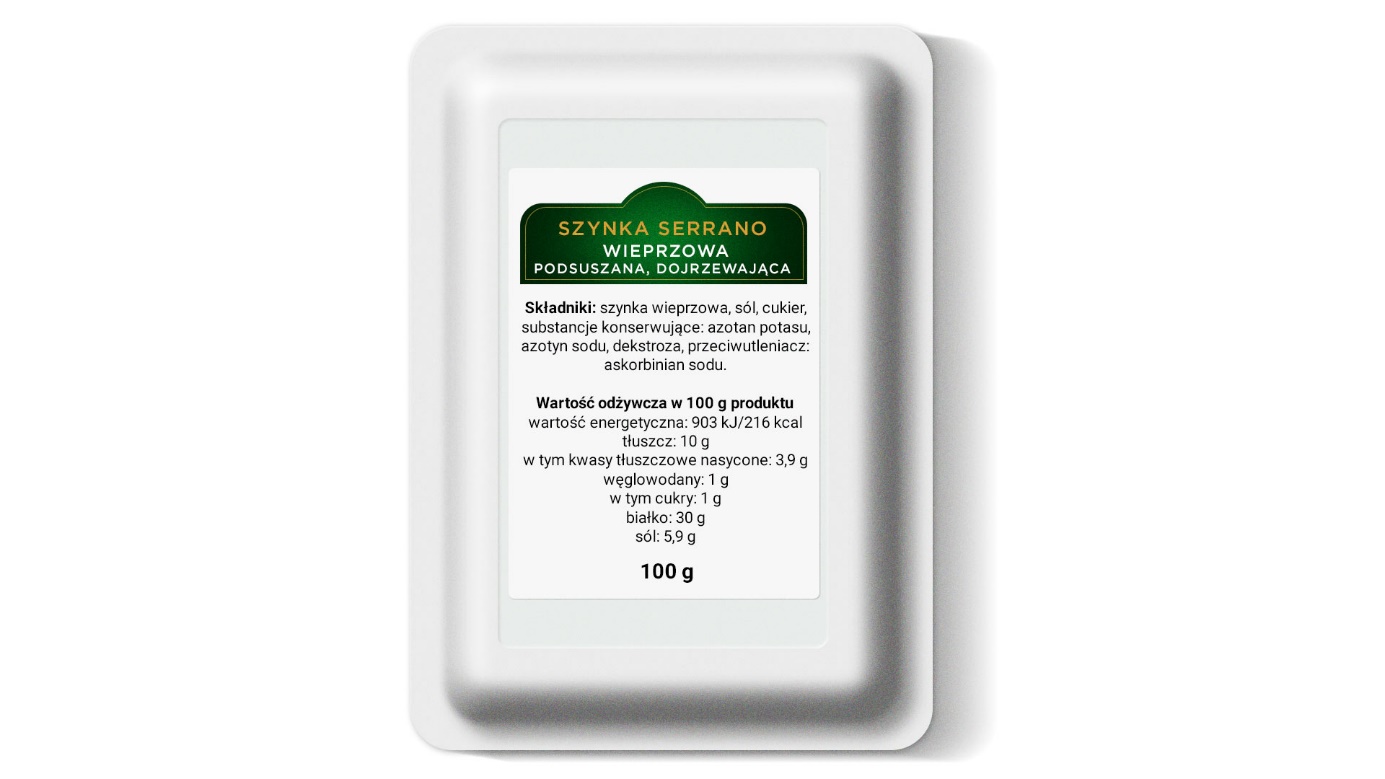 | | |
| 16 | Pork ham | C – Yellow |
| 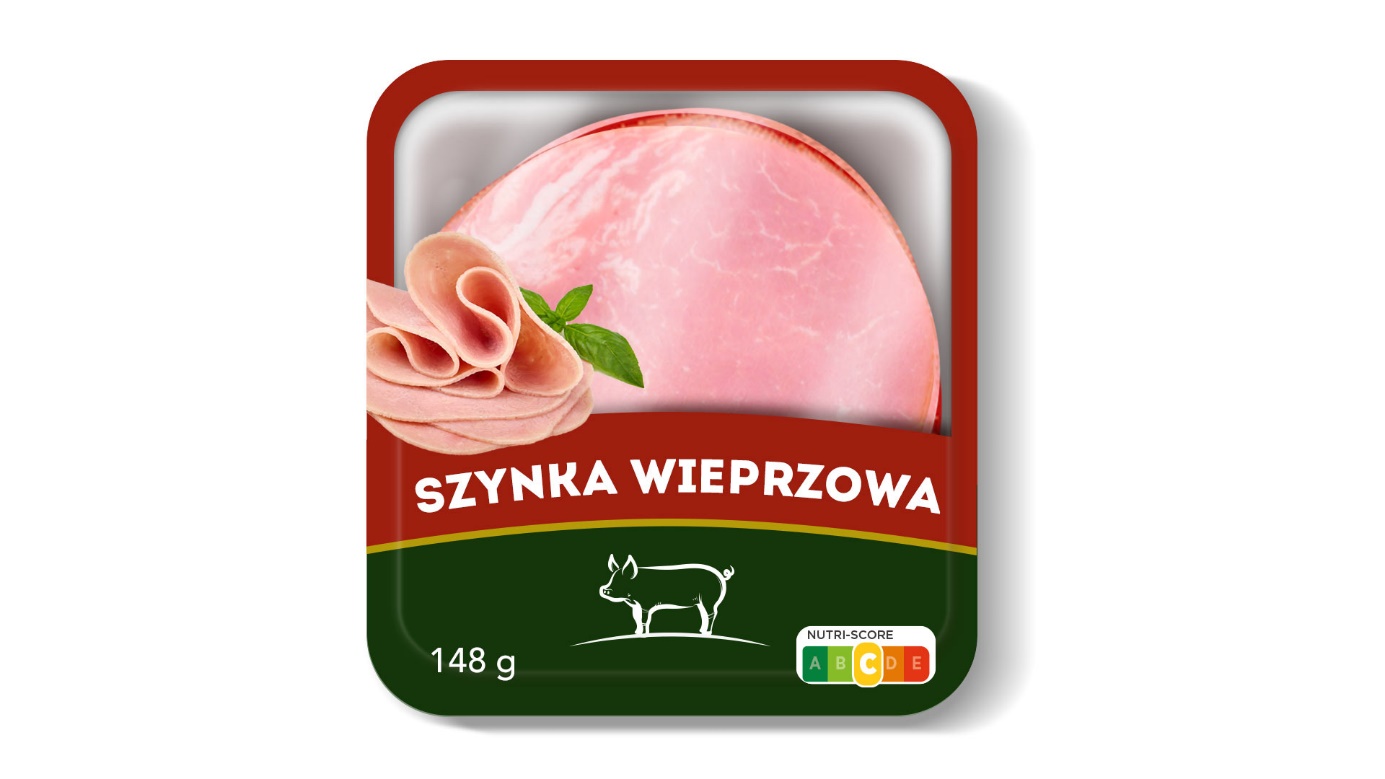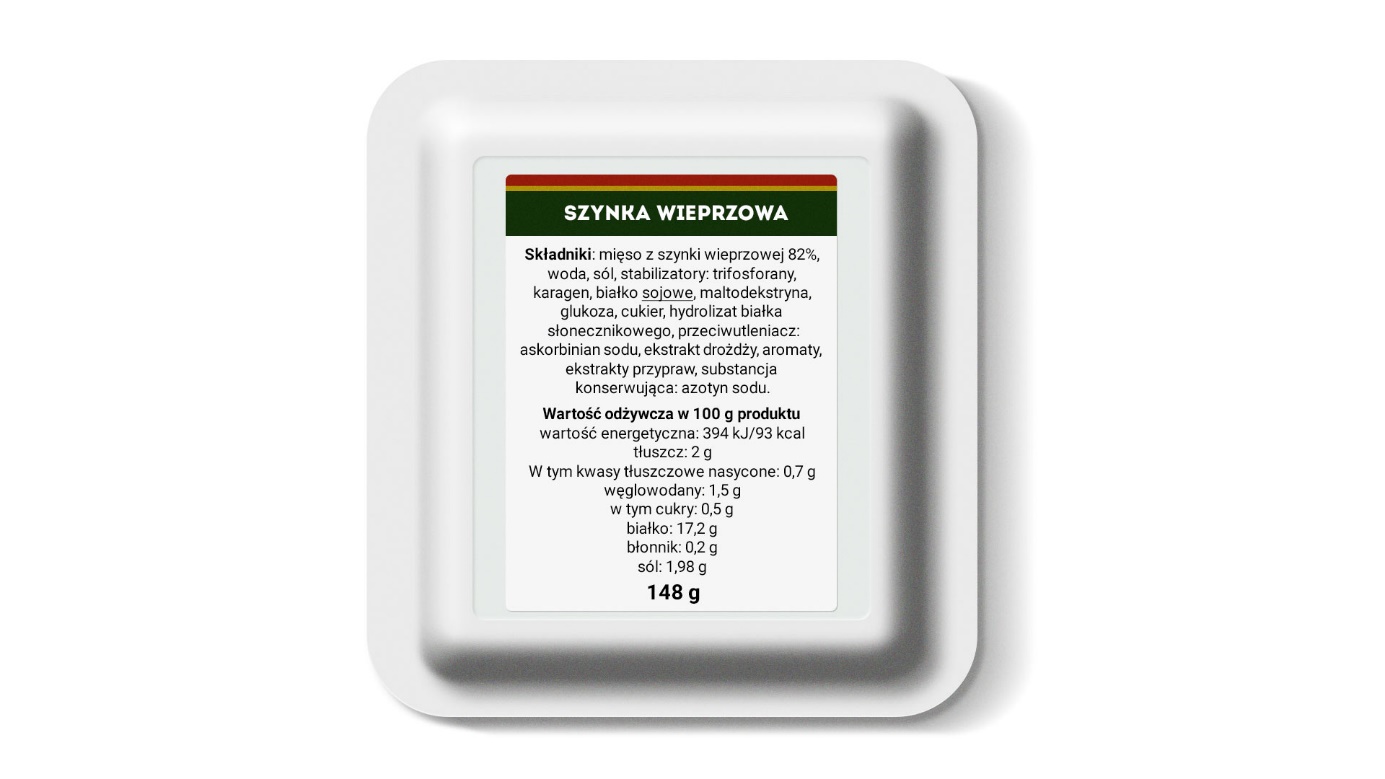 | | |
